# Convergent and divergent brain structural and functional abnormalities associated with developmental dyslexia

**DOI:** 10.7554/eLife.69523

**Published:** 2021-09-27

**Authors:** Xiaohui Yan, Ke Jiang, Hui Li, Ziyi Wang, Kyle Perkins, Fan Cao

**Affiliations:** 1 Department of Psychology, Sun Yat-Sen University Guangzhou China; 2 Department of Preschool Education, Anyang Preschool Education College Anyang China; 3 School of Foreign Language, Jining University Jining China; 4 Florida International University (Retired Professor) Miami United States; Universitat de Barcelona Spain; National Institute of Mental Health, National Institutes of Health United States

**Keywords:** dyslexia, multimodal meta-analysis, alphabetic language, morpho-syllabic language, Human

## Abstract

Brain abnormalities in the reading network have been repeatedly reported in individuals with developmental dyslexia (DD); however, it is still not totally understood where the structural and functional abnormalities are consistent/inconsistent across languages. In the current multimodal meta-analysis, we found convergent structural and functional alterations in the left superior temporal gyrus across languages, suggesting a neural signature of DD. We found greater reduction in grey matter volume and brain activation in the left inferior frontal gyrus in morpho-syllabic languages (e.g. Chinese) than in alphabetic languages, and greater reduction in brain activation in the left middle temporal gyrus and fusiform gyrus in alphabetic languages than in morpho-syllabic languages. These language differences are explained as consequences of being DD while learning a specific language. In addition, we also found brain regions that showed increased grey matter volume and brain activation, presumably suggesting compensations and brain regions that showed inconsistent alterations in brain structure and function. Our study provides important insights about the etiology of DD from a cross-linguistic perspective with considerations of consistency/inconsistency between structural and functional alterations.

## Introduction

Individuals with developmental dyslexia (DD) encounter difficulty in learning to read even with normal intelligence and adequate educational guidance (Peterson and Pennington, 2012). DD affects a large number of individuals across writing systems, and the prevalence is about 5–10% in alphabetic writing systems (e.g. English and German) (Döhla and Heim, 2015; Katusic et al., 2001; Shaywitz, 1996) and about 4–7% in morpho-syllabic writing systems (e.g. Chinese and Japanese Kanji) (Sun et al., 2013; Uno et al., 2009; Zhao et al., 2016). Multiple deficits have been identified to be associated with DD (Ring and Black, 2018), among which phonological deficit has been well-documented across languages (Gu and Bi, 2020; Snowling and Melby-Lervag, 2016). Individuals with DD show deficient phonological ability including phonological representation, manipulation, and retrieval even when compared to reading-level controls (Melby-Lervag et al., 2012; Parrila et al., 2020). However, the common phonological deficit may manifest differently in reading behavior depending on the specific requirements of the writing system. For example, phonological deficit in English is associated with lower accuracy in phonological decoding (Landerl et al., 1997; Ziegler et al., 2003), and it is associated with slower reading speed in transparent orthographies with relatively intact accuracy in phonological decoding (Wimmer and Schurz, 2010). In Chinese, phonological deficit is associated with a higher rate of semantic errors during character reading (Shu et al., 2005), because children with DD over-rely on the semantic cue in the character during reading due to the inability to use the phonological cue. According to research, 80% of Chinese characters have a semantic radical and a phonetic radical providing semantic cues and phonological cues of the character, respectively (Honorof and Feldman, 2006).

At the neurological level, the reading network in the left hemisphere has often been found to show alterations in individuals with DD (Pugh et al., 2000; Richlan, 2012; Richlan, 2014), including the temporoparietal cortex (TP), the occipitotemporal cortex (OT), and the inferior frontal cortex. The left TP area is further subdivided into the posterior superior temporal gyrus (STG), which is involved in fine phonological analysis (Petersen and Fiez, 1993; Richlan, 2012) and the inferior parietal lobule (IPL) which is associated with general attention control (Richlan, 2014). This TP region tends to show reduced brain activation in individuals with DD in alphabetic languages as demonstrated in a cross-linguistic study of English, Italian, and French (Paulesu et al., 2001) and several meta-analysis studies in alphabetic languages (Maisog et al., 2008; Martin et al., 2016; Paulesu et al., 2014; Richlan et al., 2009; Richlan et al., 2011). The left OT area, including the middle occipital gyrus (MOG), inferior temporal gyrus (ITG) and fusiform gyrus, has been consistently found to show reduced activation in individuals with DD across morpho-syllabic and alphabetic languages (Bolger et al., 2008; Cao et al., 2020; Centanni et al., 2019; Chyl et al., 2019; Paz-Alonso et al., 2018). This region is associated with visuo-orthographic processing during reading (Glezer et al., 2016; Glezer et al., 2019). The left inferior frontal cortex is further subdivided into the inferior frontal gyrus (IFG) and the precentral gyrus (Richlan, 2014). The precentral gyrus may relate to compensatory articulatory processes in dyslexia (Hancock et al., 2017), whereas the IFG has been known to be involved in phonological and semantic retrieval, lexical selection and integration (Booth et al., 2007a; Booth et al., 2007b; Costafreda et al., 2006; Szatkowska et al., 2000). However, the nature of dysfunction in the left IFG in individuals with DD remains controversial. Although reduced activation in the left IFG was confirmed by many fMRI studies and meta-analysis studies (Booth et al., 2007a; Cao et al., 2006; Richlan et al., 2010; Wimmer et al., 2010), increased activation in the left IFG was also reported in many fMRI studies (Grunling et al., 2004; Kronbichler et al., 2006; Waldie et al., 2013; Wimmer et al., 2010). The inconsistent results may be related to task and task difficulty (Waldie et al., 2013; Wimmer et al., 2010), orthographic transparency (Martin et al., 2016), and age of participants (Chyl et al., 2019).

There is a sparsity in research investigating whether the deficits associated with DD are language-universal. Paulesu et al., 2001 found that readers with DD in English, Italian, and French showed similar brain abnormality during an explicit word reading task and an implicit reading task. Hu et al., 2010 found that Chinese and English children with DD showed language-universal deficits. Feng et al., 2020 found that children with DD in both Chinese and French showed common reduction of brain activation in the left fusiform gyrus and STG. In summary, meta-analytic studies should make a greater contribution in such a topic by gathering studies from different languages and comparing them.

Even though language-universal deficits in the brain have been suggested in several studies (Feng et al., 2020; Hu et al., 2010; Paulesu et al., 2001), language specificity has been demonstrated as well (Martin et al., 2016; Siok et al., 2004). In a meta-analysis study (Martin et al., 2016), researchers directly compared brain deficits associated with DD between transparent and opaque orthographies and found that functional abnormalities in the brain vary with orthographic depth in alphabetic languages. Specifically, consistent reduction of brain activation was found in a left OT area regardless of orthographic depth, whereas greater reduction was found in the left fusiform gyrus, left TP and left IFG pars orbitalis in transparent orthographies than in opaque orthographies, and greater reduction in the bilateral intraparietal sulcus, left precuneus and left IFG pars triangularis was found in opaque orthographies than in transparent orthographies. In a recent study on Chinese-English bilingual children with DD, researchers also found both language-universal and language-specific deficits for Chinese and English (Cao et al., 2020). These findings suggest that there are both language-universal and language-specific deficits across languages. The language-universal deficits might be related to the causal risk of DD while the language-specific deficits tend to be interpreted as a result of interaction between DD and the specific language system that one studies.

Alphabetic and morpho-syllabic languages make a contrastive cross-linguistic comparison. As a representative morpho-syllabic language, Chinese character represents a morpheme and a syllable rather than a phoneme, even though a small percent of Chinese characters are logographic. Research on DD in Chinese has revealed different patterns of brain abnormalities from alphabetic languages. Significant alteration in the left middle frontal gyrus (MFG) or dorsal IFG has been consistently reported in different studies (Cao et al., 2017; Cao et al., 2020; Liu et al., 2012; Liu et al., 2013a; Siok et al., 2004; Siok et al., 2008), while the alteration in the left TP areas has been reported in only a few studies (Cao et al., 2017; Cao et al., 2018; Hu et al., 2010). This might be because the left dorsal IFG plays an essential role in Chinese. It has been found that the left dorsal IFG is more involved in Chinese reading than in English reading, while the left TP is more involved in English reading than in Chinese reading in typical readers (Bolger et al., 2005; Tan et al., 2005). Therefore, the greater deficit in the left dorsal IFG suggests a Chinese-specific deficit.

In addition to functional studies, there have also been a large number of studies with a focus on structural alterations associated with DD. Even though brain structural alterations may cause DD, it is equally possible that altered brain structure is a result of being DD, since learning experience shapes brain development. However, only one of these studies has taken language difference into account (Silani et al., 2005). Previous meta-analysis studies on alphabetic languages have found grey matter reduction in the left TP area (Linkersdörfer et al., 2012; McGrath and Stoodley, 2019; Richlan et al., 2013) as well as the left OT area (Linkersdörfer et al., 2012). These three meta-analytic studies echo findings from functional studies by showing abnormal brain structures within the classic reading network in alphabetic languages. In consistent, studies on morpho-syllabic languages have also found grey matter reduction within the classic reading network (Liu et al., 2013b; Siok et al., 2008; Wang et al., 2019; Xia et al., 2016). However, there are also studies that found abnormal brain structures outside the classic reading network, for example, in putamen, cerebellum, thalamus, and caudate etc. (Adrian-Ventura et al., 2020; Brambati et al., 2004; Brown et al., 2001; Jagger-Rickels et al., 2018; Jednorog et al., 2015; Wang et al., 2019), suggesting that these regions are also affected in this condition. Taken together, both classic reading regions and other regions have been found to show structural alterations in DD, and the previous inconsistent findings in brain structure might be due to the lack of differentiation in participants’ language. It is important to differentiate language-universal structural alterations as a core deficit which might be related to the cause of DD and language-specific structural alterations as a consequence of being DD in a specific language. Learning a specific language with DD may affect brain development in regions that are specifically important for that language and related functions (Mechelli et al., 2004).

DD is associated with altered brain structure and function, but very few studies have investigated whether brain structural and functional alterations are consistent or inconsistent. In a study by Siok et al., 2008, researchers examined both structural and functional alterations in Chinese children with DD, and found reduced GMV and brain activation in the left MFG, which underscores the association between the left MFG and DD in Chinese. Another study located a key region in the left IPL, which showed reduced GMV and activation in English-speaking readers with DD (Hoeft et al., 2007). A recent study, from a developmental perspective, found a dissociation between brain structural development and brain functional development in some brain regions (e.g. left fusiform gyrus) in the context of reading development (Siok et al., 2020), suggesting that learning experience may significantly shape brain function independent of brain structure. However, more research is sorely needed to examine whether there are brain regions that show increased GMV but decreased brain function or vice versa, and to understand the neurocognitive implications of such patterns. Simultaneously considering structural and functional abnormalities with a focus on cross-linguistic comparison would provide a comprehensive perspective to understand the neural mechanisms of DD.

In this meta-analysis study, we aimed to explore how structural and functional impairment of DD converge or diverge and whether this pattern is similar or different across writing systems. We expected to find brain regions that show decreased brain structure and function, indicating insufficient neuronal resources for certain cognitive computations. For regions that show increased brain structure and function, we believe they develop to an unusually high degree for compensation. For brain regions with increased structure but decreased function or decreased structure and increased function, it may be due to brain structures receiving inhibitory input from other regions. We also expected to find language-universal as well as language-specific neurological abnormalities. For language-universal deficits, we tend to believe that they are related to the cause of DD, while the language-specific deficits tend to be consequences of DD in different languages.

## Results

### Description of the included studies

For the functional studies, a total of 2728 participants (controls:1370, DD:1358) were included, and the mean age was 16.56 years for controls and 16.26 years for participants with DD. Specifically, there were 79 functional experiments in alphabetic languages, including 31 experiments on adults (N = 434, mean age = 26.12 for controls, N = 411, mean age = for 25.86 for DD), 36 experiments on children (N = 553, mean age = 10.59 for controls, N = 586, mean age = for 10.54 for DD), 7 experiments on adolescents (N = 131, mean age = 14.44 for controls, N = 108, mean age = for 14.30 for DD), and 5 studies of mixed ages. There were 12 functional experiments in morpho-syllabic languages (N = 164, mean age = 11.48 for controls, N = 162, mean age = for 11.45 for DD), including 11 experiments on children and 1 experiment on adolescents.

For the structural studies, there were 21 experiments in alphabetic languages, including 10 experiments on adults (N = 209, mean age = 26.68 for controls, N = 193, mean age = for 27.15 for DD), 8 experiments on children (N = 245, mean age = 10.17 for controls, N = 266, mean age = for 10.28 for DD), 1 experiment on adolescents and 2 studies of mixed ages. There were six structural experiments on children in morpho-syllabic languages (N = 89, mean age = 11.82 for controls, N = 94, mean age = for 11.74 for DD).

### Meta-analysis results

#### Functional deficits in alphabetic languages and morpho-syllabic languages

**Figure 1.**
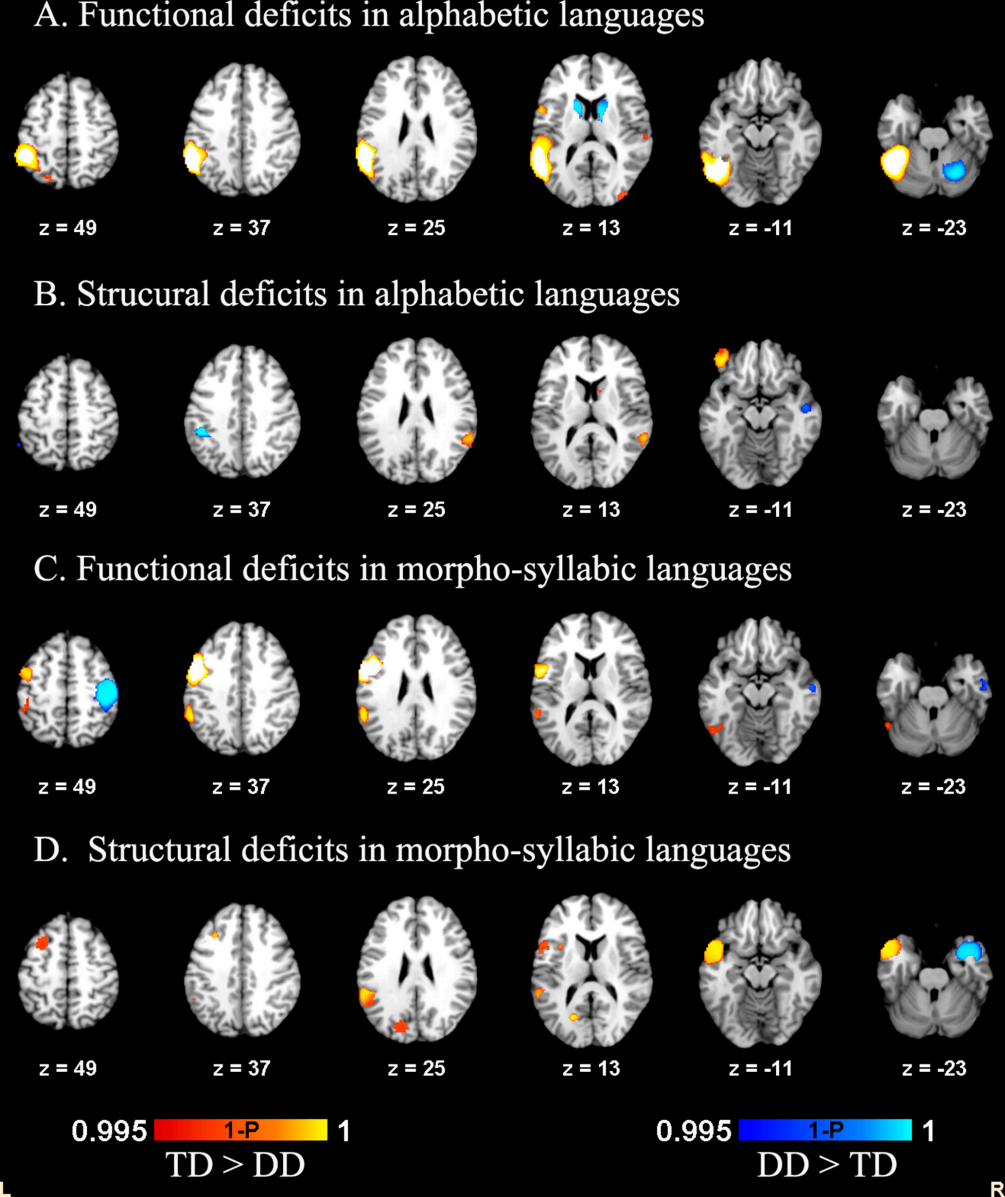
Functional and structural deficits related to DD in alphabetic languages and morpho-syllabic languages.

In the meta-analysis of functional studies in alphabetic languages, hypoactivation in DD was found in a large cluster peaked at the left supramarginal gyrus which extended to the inferior frontal cortex, occipitotemporal cortex and cerebellum, a cluster peaked at the right MOG and a cluster peaked at right STG (Table 1 and Figure 1A). Hyperactivation in DD was found in the right cerebellum and bilateral caudate nucleus.

**Table 1.** Functional deficits in individuals with DD in alphabetic languages (JK represents the results of jack-knife sensitivity analysis).

Regions	MNI coordinate	SDM-Z	p	Voxels	Cluster breakdown (Voxels)	JK
*Hypoactivation in DD*						
Left supramarginal gyrus	−56,–46,30	4.919	0.0000	10,742	Left IPL, BA 40 (986)Left MTG, BA 21 (719)Left MTG, BA 37 (634)Left ITG, BA 37 (601)Left fusiform gyrus, BA 37 (479)Left ITG, BA 20 (414)Left STG, BA 48 (377)Left supramarginal gyrus, BA 48 (329)Left angular gyrus, BA 39 (307)Left MTG, BA 22 (300)Left cerebellum, lobule VI, BA 37 (285)Left STG, BA 42 (281)Left rolandic operculum, BA 48 (261)Left arcuate network (255)Left cerebellum, crus I, BA 37 (210)Left STG, BA 22 (201)Left supramarginal gyrus, BA 40 (198)Left superior longitudinal fasciculus III (182)Left IPL, BA 2 (167)Left inferior occipital gyrus, BA 19 (156)	79/79
Right MOG	42,–86,6	2.244	0.0002	361	Right MOG, BA19 (208)	76/79
Right STG	60,–16,4	1.936	0.0011	358		73/79
*Hyperactivation in DD*						
Right cerebellum	26,–60,–28	–1.526	0.0000	1,559	Right cerebellum, lobule VI, BA 37 (352)Right cerebellum, lobule VI, BA 19 (233)Middle cerebellar peduncles (212)	79/79
Left caudate nucleus	–16,12,6	–1.459	0.0000	611	Left anterior thalamic projections (364)	79/79
Right caudate nucleus	10,2,14	–1.317	0.0001	520	Right anterior thalamic projections (185)Right caudate nucleus (184)	79/79

In the meta-analysis of functional studies in morpho-syllabic languages, hypoactivation in DD was found in left IFG opercular part, left supramarginal gyrus and left ITG. Hyperactivation in DD was found in right precentral gyrus and right middle temporal gyrus (MTG) (Table 2, Table 3 and Figure 1C). The jack-knife sensitivity analysis showed that all results reported above were replicable (Table 1; Table 3).

**Table 2.** Structural deficits in individuals with DD in alphabetic languages (JK represents the results of jack-knife sensitivity analysis).

Regions	MNI coordinate	SDM-Z	p	Voxels	Cluster breakdown (Voxels)	JK
*Decreased GMV in DD*						
Left IFG orbital part	−38,42,–16	2.306	0.0001	611	Left IFG orbital part, BA 47 (217)	20/21
Right STG	56,–44,18	2.024	0.0003	560	Right STG, BA 42 (156)	20/21
Right caudate	6,14,2	1.695	0.0022	166		21/21
*Increased GMV in DD*						
Left IPL	−42,–36,36	–1.976	0.0000	237	Left IPL, BA 40 (237)	20/21
Right MTG	50,–12,–14	–1.040	0.0014	174		21/21

**Table 3.** Functional deficits in individuals with DD in morpho-syllabic languages (JK represents the results of jack-knife sensitivity analysis).

Regions	MNI coordinate	SDM-Z	p	Voxels	Cluster breakdown (Voxels)	JK
*Hypoactivation in DD*						
Left IFG opercular part	–48,10,28	4.071	0.0000	2527	Left precentral gyrus, BA 6 (623)Left IFG opercular part, BA 44 (278)Left precentral gyrus, BA 44 (195)Corpus callosum (178)Left MFG, BA 44 (162)	12/12
Left supramarginal gyrus	−58,–42,26	2.149	0.0001	1001	Left IPL, BA 40 (271)Left STG, BA 42 (153)Left supramarginal gyrus, BA 48 (144)	11/12
Left ITG	−48,–56,–18	1.761	0.0008	326	Left ITG, BA37 (166)	9/12
*Hyperactivation in DD*						
Right precentral gyrus	52,–16,44	–2.035	0.0000	2201	Right precentral gyrus, BA 6 (640)Right postcentral gyrus, BA 3 (447)Right precentral gyrus, BA 4 (350)Right postcentral gyrus, BA 4 (215)	12/12
Right MTG	56,–10,–18	–1.453	0.0013	298		10/12

#### Structural deficits in alphabetic languages and morpho-syllabic languages

In the meta-analysis of structural studies in alphabetic languages, readers with DD showed a decrease in GMV in the left IFG orbital part, right STG and right caudate nucleus (Table 2 and Figure 1B). In contrast, readers with DD showed an increase in GMV in the left IPL and right MTG. In the meta-analysis of structural studies in morpho-syllabic languages, readers with DD showed a decrease in GMV in the left temporoparietal cortex, left calcarine cortex and left MFG. Readers with DD showed an increase in GMV in the right STG (Table 4 and Figure 1D). The jack-knife sensitivity analysis showed that all results reported were replicable (Table 2; Table 4).

**Table 4.** Structural deficits in individuals with DD in morpho-syllabic languages (JK represents the results of jack-knife sensitivity analysis).

Regions	MNI coordinate	SDM-Z	p	Voxels	Cluster breakdown (Voxels)	JK
*Decreased GMV in DD*						
Left STG	−50,4,–4	2.466	0.0000	2,948	Left insula, BA 48 (539)Left STG, BA 38 (392)Left rolandic operculum, BA 48 (226)Left MTG, BA 21 (215)Left STG, BA 48 (186)	6/6
Left temporoparietal cortex	−56,–40,18	2.102	0.0002	900	Left supramarginal gyrus, BA 48 (188)Left STG, BA 42 (171)	6/6
Left calcarine cortex	−20,–66,14	2.447	0.0000	449	Corpus callosum (297)	6/6
Left MFG	–32,26,40	2.319	0.0001	438		6/6
*Increased GMV in DD*						
Right STG	34,6,–26	–1.572	0.0001	1,829	Right STG, BA 38 (261)Right ITG, BA 20 (250)Right MTG, BA 20 (212)	6/6
Right precuneus	12,–52,42	–1.254	0.0014	156		2/6

In the supplementary materials, we reported whether the structural and functional deficits found in the current study were reported in each study included in the meta-analysis (Supplementary file 1f-1i).

#### Comparison between alphabetic and morpho-syllabic languages

**Figure 2.**
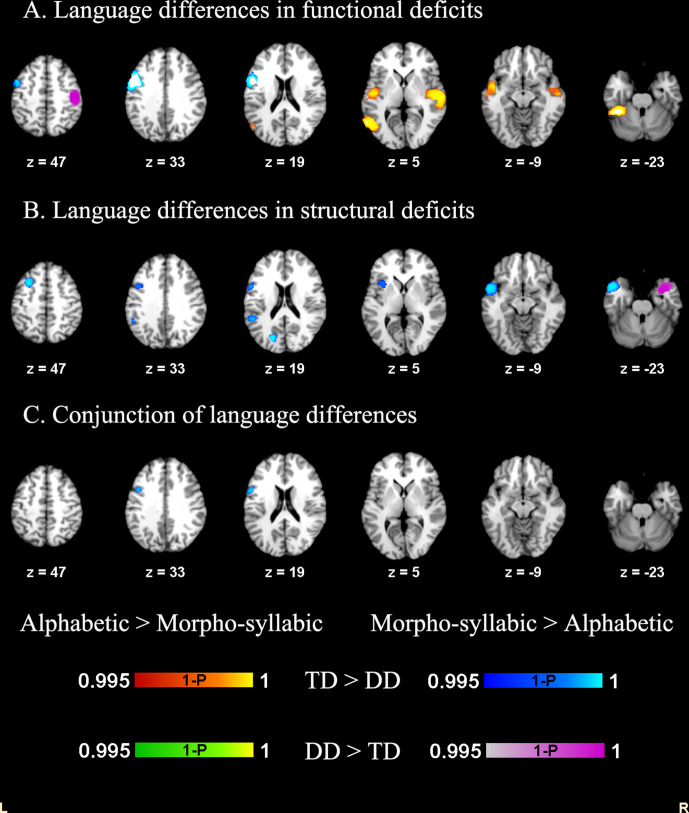
Direct comparisons between the alphabetic group and the morpho-syllabic group in structural and functional deficits. Conjunction analysis showed greater reduction of both GMV and brain activation in the left dorsal IFG in morpho-syllabic languages than alphabetic languages.

For the direct comparison between the morpho-syllabic and alphabetic groups in functional studies, we found greater reduction of brain activation in alphabetic languages than in morpho-syllabic languages in the left MTG, right STG and left fusiform gyrus. We found greater reduction of brain activation in morpho-syllabic languages than in alphabetic languages in the left IFG, opercular part and greater increase of brain activation in DD in morpho-syllabic languages than in alphabetic languages in the right precentral gyrus (Table 5, Figure 2A).

**Table 5.** Direct comparisons between alphabetic languages and morpho-syllabic languages in functional studies.

Regions	MNI coordinate	SDM-Z	p	Voxels	Cluster breakdown (Voxels)
*Hypoactivation in DD*					
*Alphabetic languages > Morpho-syllabic languages*					
Left MTG	−54,–62,8	1.203	0.0000	2,173	Left MTG, BA 37 (453)Left MTG, BA 48 (294)Left MTG, BA 21 (293)Corpus callosum (184)
Right STG	60,–18,4	1.080	0.0000	2047	Right STG, BA 22 (400)Corpus callosum (373)Right insula, BA 48 (278)Right STG, BA 48 (259)Right rolandic operculum, BA 48 (193)
Left fusiform gyrus	−40,–42,–24	1.279	0.0000	924	Left fusiform gyrus, BA 37 (198)Left ITG, BA 20 (198)
*Morpho-syllabic languages > Alphabetic languages*					
Left IFG opercular part	–48,8,30	–3.945	0.0000	2093	Left precentral gyrus, BA 6 (512)Left IFG opercular part, BA 44 (274)Left precentral gyrus, BA 44 (191)Corpus callosum (161)Left IFG, triangular part, BA 48 (159)
*Hyperactivation in DD*					
*Morpho-syllabic languages > Alphabetic languages*					
-	-	-	-	-	-
*Morpho-syllabic languages > Alphabetic languages*					
Right precentral gyrus	40,–20,54	–2.262	0.0000	1,518	Right precentral gyrus, BA 6 (525)Right precentral gyrus, BA 4 (306)Right postcentral gyrus, BA 3 (286)Right postcentral gyrus, BA 4 (171)

**Figure 2—figure supplement 1.**
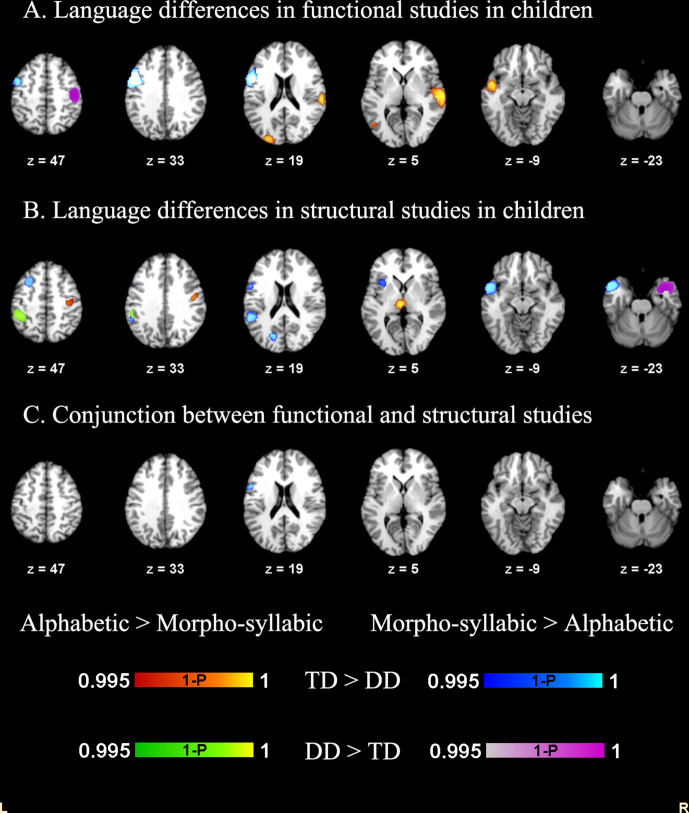
Language differences in functional studies and structural studies in children.

**Figure 2—figure supplement 2.**
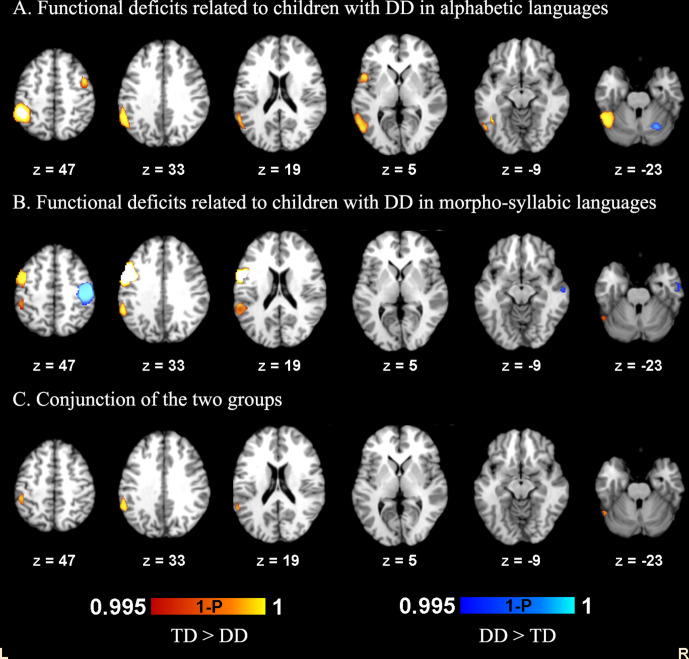
Functional deficits in children with DD in each group and common deficits between them.

**Figure 2—figure supplement 3.**
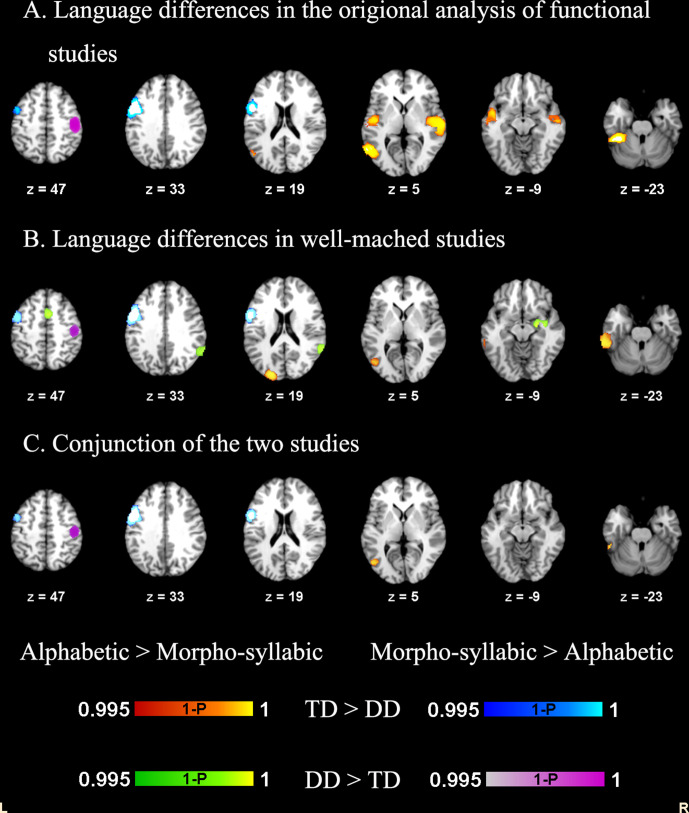
Language differences between the two well-matched groups in the confirmation analysis and how the current results overlap with the original results.

For the direct comparison between the morpho-syllabic and alphabetic groups in structural studies, we found greater reduction of GMV in DD in morpho-syllabic languages than in alphabetic languages in the left STG, left IFG opercular part, left MFG, left supramarginal gyrus, left superior occipital gyrus (SOG) and left insula. We also found greater increase of GMV in DD in morpho-syllabic languages than in alphabetic languages in the right STG and left ITG (Table 6, Figure 2B). We found no regions that showed greater GMV alterations in alphabetic languages than in morpho-syllabic languages.

**Table 6.** Direct comparisons between alphabetic languages and morpho-syllabic languages in structural studies.

Regions	MNI coordinate	SDM-Z	P	Voxels	Cluster breakdown (Voxels)
*Decreased GMV in DD*					
*Alphabetic languages > Morpho-syllabic languages*					
-	-	-	-	-	-
*Morpho-syllabic languages > Alphabetic languages*					
Left STG	−50,10,–18	–3.139	0.0000	1,218	Left STG, BA 38 (322)
Left IFG opercular part	–52,10,24	–2.253	0.0008	409	
Left MFG	–28,16,44	–2.888	0.0001	346	
Left supramarginal gyrus	−48,–44,24	–2.761	0.0001	344	
Left SOG	−20,–72,20	–2.911	0.0001	277	Corpus callosum (187)
Left insula	–32,14,8	–2.046	0.0015	179	
*Increased GMV in DD*					
*Alphabetic languages > Morpho-syllabic languages*					
-	-	-	-	-	-
*Morpho-syllabic languages > Alphabetic languages*					
Right STG	34,6,–28	–1.893	0.0001	1,397	Right STG, BA 38 (246)
Left ITG	−54,–58,–16	–1.112	0.0018	161	

To identify the common language differences between the structural and functional studies, we conducted a conjunction analysis between the language differences in structural studies and functional studies. This produced an overlap of 377 voxels in the left IFG opercular part, with a peak at (–52, 10, 24), indicating greater reduction of both GMV and brain activation in morpho-syllabic languages than in alphabetic languages (Figure 2C).

#### Multimodal analysis results in alphabetic and morpho-syllabic languages

**Figure 3.**
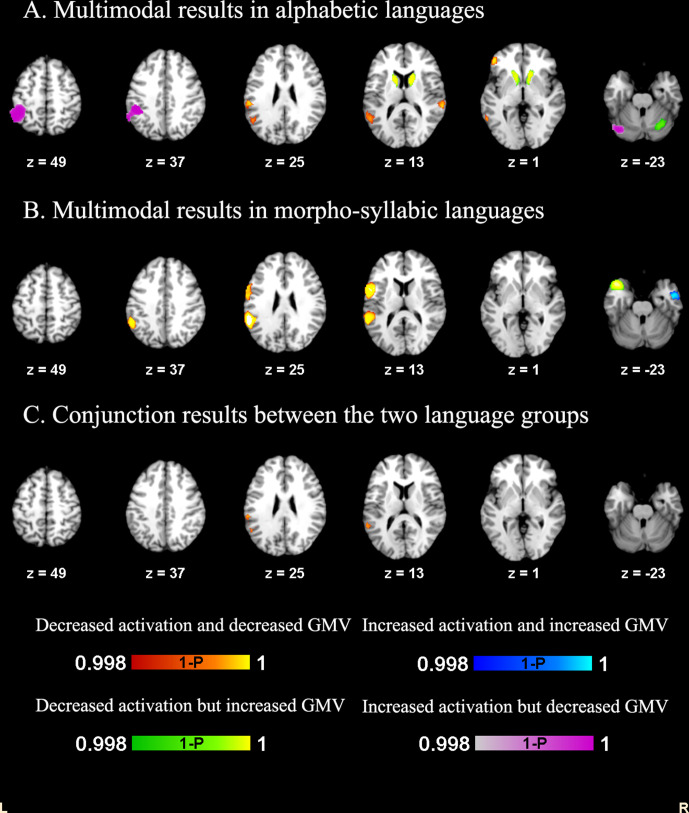
Structural and functional deficits in DD for alphabetic languages and morpho-syllabic languages. Decreased GMV and brain activation were found in both groups in the left STG.

Multimodal meta-analysis in alphabetic languages showed that decreased GMV and hypoactivation in DD were found in the bilateral STG and left IFG triangular part; no regions showed increased GMV and hyperactivation; increased GMV and hypoactivation in DD were found in left IPL and left cerebellum; decreased GMV and hyperactivation in DD were found in bilateral caudate and right cerebellum (Table 7, Figure 3A). Multimodal meta-analysis in morpho-syllabic languages showed that decreased GMV and hypoactivation in DD were found in the left STG and left IFG opercular part; increased GMV and hyperactivation in DD were found in the right MTG; decreased GMV and hyperactivation in DD were found in left STG; no regions showed increased GMV and hypoactivation (Table 8, Figure 3B).

**Table 7.** Multimodal structural and functional abnormalities in individuals with DD in alphabetic languages.

Regions	MNI coordinate	Voxels	Cluster breakdown (Voxels)
*Decreases of GMV and hypoactivation in DD*			
Left STG	−52,–30,20	1,099	Left MTG, BA 21 (237)
Right STG	62,–32,14	322	
Left IFG, triangular part	–46,42,0	219	
*Increases of GMV and hypoactivation in DD*			
Left IPL	−46,–40,38	1,689	Left IPL, BA 40 (941)
Left cerebellum	−40,–70,–24	446	Left cerebellum, crus I, BA 19 (159)
*Decreases of GMV and hyperactivation in DD*			
Right cerebellum	28,–52,–34	1,286	Right cerebellum, lobule VI, BA 37 (273)Middle cerebellar peduncles (267)Right cerebellum, lobule VI, BA 19 (204)
Right caudate	8,8,12	600	Right anterior thalamic projections (214)Right caudate nucleus (189)
Left caudate	–16,8,14	595	Left anterior thalamic projections (353)

**Table 8.** Multimodal structural and functional abnormalities in individuals with DD in morpho-syllabic languages.

Regions	MNI coordinate	Voxels	Cluster breakdown (Voxels)	
*Decreases of GMV and hypoactivation in DD*				
Left STG	−58,–38,22	1,566	Left supramarginal gyrus, BA 48 (254)Left STG, BA 42 (253)Left supramarginal gyrus, BA 40 (153)	
Left IFG opercular part	–56,2,10	1,052	Left IFG opercular part, BA 44 (151)	
*Decreases of GMV and hyperactivation in DD*				
Left STG	−44,16,–22	854	Left STG, BA 38 (394)	
*Increases of GMV and hyperactivation in DD*				
Right MTG	48,–6,–26	493	Right MTG, BA 21 (178)	
				

To identify the common multimodal deficits in alphabetic languages and morpho-syllabic languages, we conducted a conjunction analysis of the thresholded multimodal maps of the two types of writing systems. This procedure produced an overlap of 482 voxels in the left STG, which peaked at (−54,–34, 20), indicating shared reduction of GMV and hypoactivation in both types of writing systems (Figure 3C).

#### Confirmation analysis results

For the confirmation analysis on children only in the alphabetic group, when we compared children in alphabetic languages to children in morpho-syllabic languages, we found a conjunction of the language differences in the functional studies and the structural studies, which was greater reduction of brain activation and GMV in morpho-syllabic languages than in alphabetic languages in the left IFG opercular part (–54, 10, 20) with a cluster of 273 voxels (Figure 2—figure supplement 1C), which is consistent with the original result. Conjunction analysis of the functional alterations in the alphabetic group and the morpho-syllabic group revealed common reduction of brain activation in children with DD in the left ITG (−48,–56, –18) and the left TP area (−56,–44, 32) with a cluster size of 291 voxels and 778 voxels, respectively. The TP area overlapped with the original multimodal result at the left STG (−56,–48, 22) where both language groups showed reduced brain activation and GMV. Taken together, these results are consistent with the original results, suggesting that the language differences are not due to unmatched age range. For detailed results of the confirmation analysis, please see Supplementary file 1a-1d, Figure 2—figure supplement 1, Figure 2—figure supplement 2.

Furthermore, the other confirmation analysis on functional studies of two well-matched subgroups on age, task and number of studies also confirmed our original findings. Conjunction analysis between the original results and the current functional differences between English and Chinese showed consistent greater reduction of brain activation in DD in morpho-syllabic languages/Chinese than in alphabetic languages/English in the left dorsal IFG (–52, 6, 16) with a cluster size of 1966 voxels. There was consistent greater reduction of brain activation in DD in alphabetic languages/English than in morpho-syllabic languages/Chinese in the left MTG (−48,–68, 4) and left MOG (−51,–41, –24) with a cluster size of 166 voxels and 90 voxels, respectively. Consistent greater increase of brain activation in morpho-syllabic languages/Chinese than in alphabetic languages/English was also found in the right precentral gyrus (44, -22, 50), and the cluster size was 792 voxels (Figure 2—figure supplement 3C). For detailed results of this confirmation analysis, please see Supplementary file 1e, Figure 2—figure supplement 3.

## Discussion

In this meta-analysis study, we examined the convergence and divergence between the brain structural and functional deficits associated with DD as well as whether the deficits are consistent across languages. We found that readers with DD showed both GMV reduction and functional hypoactivation in the left TP and ventral IFG in alphabetic languages, readers with DD showed both GMV reduction and functional hypoactivation in the left TP and dorsal IFG in morpho-syllabic languages, among which, the left STG was a shared impairment across all languages, and the dorsal left IFG showed a greater impairment in morpho-syllabic languages than in alphabetic languages, suggesting both language-universal and language-specific deficits in the brain. We also found GMV increase and functional hyperactivation in the right anterior MTG/ITG region in morpho-syllabic languages; however, conjunction analysis between morpho-syllabic languages and alphabetic languages did not reveal any overlap. In addition to the consistent structural and functional alterations, we also detected inconsistent structural and functional alterations. Individuals with DD showed increased GMV and hypoactivation in the left IPL and left cerebellum, and decreased GMV and hyperactivation in the bilateral caudate in alphabetic languages, but decreased GMV and hyperactivation in left STG in morpho-syllabic languages. However, conjunction analysis between morpho-syllabic languages and alphabetic languages did not reveal any overlap.

### Convergent structural and functional impairment across writing systems

Across writing systems, convergent structural and functional deficit was found in the left STG due to reduced GMV and brain activation in both alphabetic languages and morpho-syllabic languages. Further confirmation analysis confirmed that this is a stable deficit across children and adults. This is consistent with previous meta-analysis studies (Maisog et al., 2008; McGrath and Stoodley, 2019; Paulesu et al., 2014; Richlan et al., 2013). The left STG is a very important component in the language network (Friederici, 2012; Hickok and Poeppel, 2007) as well a key region in the reading network (Pugh et al., 2000; Richlan, 2014). It is involved in phonological representation and phonological processing during both spoken language processing and reading (Bolger et al., 2005; Enge et al., 2020; Tan et al., 2005). Recent research has suggested that proficient reading is characterized by convergence between speech and print at this region regardless of languages, as multivariate brain activity patterns are similar for speech and print at this region (Chyl et al., 2021). Therefore, the reading network may develop based on the built-in language circuit, as reading is a skill that humans acquire too late in the course of human evolution to have a brain network dedicated to it. Recently, a growing number of studies have investigated early signs of dyslexia before the onset of reading and found that structural and functional deficits in the left TP area and left inferior frontal cortex appear before reading onset (Clark et al., 2014; Hosseini et al., 2013; Plewko et al., 2018; Raschle et al., 2012; Raschle et al., 2014; Vandermosten et al., 2019). It further suggests that DD might be due to early abnormality in the language network. Specifically, Skeide et al., 2018 found hypermyelination in the left auditory cortex in readers with DD using ultra-high-field MRI at 7T, and disrupted neural firing induced by hypermyelination in the layer IV of the auditory cortex, which may cause hypoactivation in the left STG. The left STG actually serves as an important hub in the language and reading network (Fernández et al., 2020), which connects the inferior frontal network and the OT network through a dorsal pathway and a ventral pathway (Brauer et al., 2013; Cummine et al., 2015). Disconnection with the left STG has been verified in task-related functional connectivity studies (Boets et al., 2013; Cao et al., 2017; Schurz et al., 2015) and a resting-state functional connectivity study (Schurz et al., 2015), as well as in a meta-analysis of DTI studies (Vandermosten et al., 2012). Our finding suggests that dyslexia is associated with structural and functional abnormalities of the left STG regardless of language. The evidence suggests that this is a neural signature of DD, which supports the phonological deficit hypothesis (Shaywitz et al., 1998).

However, we failed to find consistent structural and functional deficits in the OT area. The main reason was that there was no structural alteration but only functional reduction at this area. The OT area is a key region for orthographic recognition during visual word processing (Glezer et al., 2009; Glezer et al., 2016; Hirshorn et al., 2016; Nobre et al., 1994) and was reported to be impaired in individuals with DD (McCrory et al., 2005; Richlan et al., 2010; Wandell et al., 2012). The specialization of this region for orthographic processing is developed along with reading acquisition (Brem et al., 2010), and the dysfunction of the OT area in DD is possibly a result of reading failure (Pugh et al., 2000). A recent meta-analysis of VBM studies (McGrath and Stoodley, 2019) also failed to detect structural deficit in the OT area, which is consistent with our finding. Taken together, the lack of structural deficits with only hypoactivation at the OT area appears to suggest that the visuo-orthographic deficits at the OT might be a consequence of being DD. In contrast, the left STG which was discussed above, appears to be associated with the cause of DD. Our results provide further support for the phonological deficit hypothesis that phonological deficit is the primary deficit and other deficits may be a result of the phonological deficit (Pugh et al., 2000).

### Language differences in structural and functional alterations

The left dorsal IFG which peaked at (–52, 10, 24) showed greater reduction in both GMV and brain activation in morpho-syllabic languages than in alphabetic languages, suggesting greater impairment in this region in morpho-syllabic languages than in alphabetic languages. This finding is also verified in the confirmation analyses. Previously, many Chinese studies have reported impairment at the dorsal left IFG, for example, reduced brain activation in an auditory rhyming judgment task in children with DD at (–44, 10, 26) (Cao et al., 2017), in a lexical decision task at (–44, 3, 29) (Siok et al., 2004), in a homophone judgment task at (–55, 5, 22) (Siok et al., 2008), and in a morphological task at (–36, 8, 26) (Liu et al., 2013a). This left dorsal IFG has been believed to be more involved in Chinese reading than in alphabetic languages, with a peak at the left MFG (–46, 18, 28) as reported in a previous meta-analysis study (Tan et al., 2005). The dorsal IFG was found to be involved in phonological processing in Chinese reading (Wu et al., 2012), and it is thought to be related to addressed phonology during Chinese character reading (Tan et al., 2005). Our study adds to the literature that by direct comparison, this region does show greater deficit in individuals with DD in morpho-syllabic languages than in alphabetic languages in terms of both brain activation and GMV. This might be due to the fact that healthy Chinese readers have increased GMV and brain activation in the left dorsal IFG than healthy alphabetic readers, because the features of Chinese require greater involvement of this region in reading than alphabetic languages due to the whole-character-to-whole-syllable mapping. Actually, two cross-linguistic studies have argued that different findings of DD in different languages are actually driven by the fact that control readers show language-specific brain activation patterns (Feng et al., 2020; Hu et al., 2010), and that brain activation in individuals with DD is actually the same across languages. For example, Hu et al., 2010 found that Chinese control readers showed greater activation in the left IFG, and English control readers showed greater activation in the left superior temporal sulcus; however, children with DD in Chinese and English showed similar brain activation in these two regions. Therefore, readers with DD fail to show language specialization due to their limited reading experience and skills. In summary, this language-specific deficit is believed to be a consequence of being DD in learning morpho-syllabic languages, indicating their inability to accommodate to their own writing system.

In the direct comparison between alphabetic and morpho-syllabic languages, we also found greater hypoactivation in DD in alphabetic languages than in morpho-syllabic languages in the left MTG, right STG and left fusiform gyrus, which was verified in the well-matched confirmation analysis, suggesting that the language difference should not be due to differences in age, tasks, and number of studies in the two language groups. Our finding is consistent with previous neuroimaging studies that revealed reduced activation associated with DD in the posterior reading network in alphabetic languages (Paulesu et al., 2001; Richlan et al., 2010; Vandermosten et al., 2019), suggesting deficient orthographic and phonological processing. However, the novelty of the current study is to demonstrate greater severity of deficit in these regions in alphabetic languages than in morpho-syllabic languages. In a previous meta-analysis study of alphabetic languages, it was found that there was greater hypoactivation in the left fusiform gyrus (−40,–42, –16) in shallow orthographies than in deep orthographies (Martin et al., 2016). The explanation is that this region is associated with bottom-up rapid processing of letters, because it was found that at a proximal region (−38,–50, –16), there was a word length effect for German nonwords in non-impaired readers (Schurz et al., 2010). Moreover, the left fusiform gyrus has been found to be more involved in English reading than in Chinese reading in typical mature readers with a peak of the effect at (−44,–56, –12) (Tan et al., 2005). Therefore, the left fusiform gyrus is important for letter-by-letter orthographic recognition in alphabetic languages, and this explains why we found greater deficit in this region in alphabetic languages than in morpho-syllabic languages. As for the right STG, the previous meta-analysis found greater hypoactivation in deep orthographies than shallow orthographies (Martin et al., 2016). Together with our finding, it suggests that the right STG might be associated with the inconsistent mapping between graphemes and phonemes in deep orthographic alphabetic languages. In summary, these greater deficits in alphabetic languages than in morpho-syllabic languages might be due to the inability to adapt to the special features of alphabetic languages in individuals with DD.

For the structural studies, we found greater GMV alterations in morpho-syllabic languages than in alphabetic languages, including greater GMV reduction in the left STG, left MFG, left supramarginal gyrus, left SOG and left insula, as well as greater GMV increase in the right STG and ITG. However, considering the limited number of studies included in the morpho-syllabic language group and inconsistent results with functional studies, the results should be interpreted with caution.

### Increased GMV and hyperactivation

In the multi-modal meta-analysis, we found increased GMV and hyperactivation in participants with DD in the right MTG which was driven by the morpho-syllabic languages. For the functional studies, we also found greater hyperactivation in the right precentral gyrus in morpho-syllabic languages than in alphabetic languages. These alterations might be related to the compensation mechanism of the right hemisphere. As precentral gyri play an important role in articulation (Dronkers, 1996), overactivation in the precentral gyrus is interpreted as an articulation strategy used by individuals with DD to compensate for their deficient phonological processing (Cao et al., 2018; Shaywitz et al., 1998; Waldie et al., 2013). The compensation in the right MTG is developed in morpho-syllabic languages presumably due to the tight connection between orthography and semantics (Wang et al., 2015). Substantial evidence has shown that dyslexia was often accompanied by excessive activation of the right hemisphere (Cao et al., 2017; Cao et al., 2018; Kovelman et al., 2012; Kronschnabel et al., 2014; Yang and Tan, 2020) and reduced left lateralization of the language network (Altarelli et al., 2014; Bloom et al., 2013). Furthermore, training studies have also found increased activation in many regions in the right hemisphere in individuals with DD after reading intervention (Barquero et al., 2014; Meyler et al., 2008), suggesting the compensatory role of the right hemisphere when the left language/reading network is deficient (Coslett and Monsul, 1994; Weiller et al., 1995). However, according to the previous meta-analysis study, different regions showed overactivation in different writing systems (Martin et al., 2016). In particular, the left anterior insula showed greater overactivation in deep orthographies while the left precentral gyrus showed greater overactivation in shallow orthographies in individuals with DD. Taken together, it suggests that different compensatory mechanisms are developed depending on the characteristics of the writing system as well as learning experiences, and the compensation in the right MTG and right precentral gyrus appears to be particularly salient in morpho-syllabic languages. However, it is also possible that the increased GMV and hyperactivation in individuals with DD are due to some fundamental deficits rather than compensation. Further research is needed to understand the nature of these alterations by running brain-behavioral correlation and/or employing longitudinal designs.

### Divergent structural and functional alterations in DD

In the multimodal analysis, we also found divergent structural and functional changes related to DD, including the left IPL and left cerebellum where there was increased GMV and hypoactivation and bilateral caudate where there was reduced GMV and hyperactivation in alphabetic languages; There was decreased GMV and hyperactivation in left STG in morpho-syllabic languages. This is in line with a recent study which found a dissociation between the developmental changes of brain structure and function (Siok et al., 2020), suggesting that learning experience may sometimes shape the brain function independent of the brain structure.

### The left IPL

We found increased GMV and hypoactivation in DD in the left IPL in alphabetic languages. Consistent hypoactivation in the left IPL in DD has been documented in previous studies (Maisog et al., 2008; Martin et al., 2016). Furthermore, it was found that the deficit of the left IPL was greater in children than in adults with DD (Richlan et al., 2011), suggesting that the functional impairment of the left IPL may gradually recover with development. This may be related to the transfer of the reading circuit from the dorsal pathway to the ventral pathway over development (Younger et al., 2017). Control children activate the left IPL to a greater degree than control adults because they rely more on the dorsal pathway. Therefore, children with DD show a great reduction in the left IPL in comparison to adults with DD. Alternatively, the left IPL has been found to be deactivated during language tasks (Cao et al., 2008; Cao et al., 2017; Meyler et al., 2008; Schulz et al., 2009), and this is due to the nature of the default mode network (Laird et al., 2009), which is deactivated during active tasks. Therefore, it might be the case that the increased GMV in individuals with DD increases inhibitory inputs received by the IPL, which results in greater deactivation.

For structural studies involving the left IPL, the results are inconsistent. The GMV of the left supramarginal gyrus around the posterior part of perisylvian cortex was found to be reduced in individuals with DD (Linkersdörfer et al., 2012; McGrath and Stoodley, 2019) and it showed a positive correlation with reading accuracy only in normal readers (Jednorog et al., 2015). However, the GMV of the left inferior parietal cortex excluding the supramarginal and the angular was found to increase in individuals with DD (McGrath and Stoodley, 2019) and a study showed that the volume of the left inferior parietal cortex in control readers was negatively correlated with reading level (Houston et al., 2014). The IPL in the current study is outside the supramarginal and angular gyrus; therefore, it is consistent with the previous findings that there is increased GMV in individuals with DD.

### The cerebella

Increased GMV and hypoactivation in DD were also found in the left cerebellum in alphabetic languages; however, in the right cerebellum, we found decreased GMV and hyperactivation. Previously, it was found that the right cerebellum is greater in size than the left cerebellum in healthy controls while the asymmetry is reduced in individuals with DD (Kibby et al., 2008; Rae et al., 2002). This is consistent with our finding of increased GMV in the left cerebellum and decreased GMV in the right cerebellum in individuals with DD, suggesting reduced asymmetry in cerebellum.

The cerebella have been found to play an important role in inner speech, automatization in reading and suppression of overt articulatory movement in silent reading (Ait Khelifa-Gallois et al., 2015). Functional abnormality of cerebellum in DD has been reported repeatedly; however, hyperactivation was reported more often in the right cerebellum (Feng et al., 2017; Hernandez et al., 2013; Kronschnabel et al., 2014; Richlan et al., 2010; Rumsey et al., 1997; van Ermingen-Marbach et al., 2013a), while hypoactivation was reported more often in the left cerebellum (Christodoulou et al., 2014; McCrory et al., 2000; Olulade et al., 2012; Reilhac et al., 2013; Siok et al., 2008). This is consistent with our finding of hyperactivation in the right cerebellum and hypoactivation in the left cerebellum. Different alteration patterns in the left and right cerebellum suggest that they may play different roles in reading and dyslexia. The right cerebellum has been found to be connected with the left frontal-parietal pathway for phonological processing and with the left frontal-temporal pathway for semantic processing (Alvarez and Fiez, 2018; Gatti et al., 2020). The left cerebellum, however, is involved in error monitoring during reading unfamiliar non-words (Ben-Yehudah and Fiez, 2008), as well as articulation related movement process, since it is activated in reading aloud but not in lexical decision (Carreiras et al., 2007). Richards et al., 2006 argued that the left cerebellum is involved in processing the morphology of word forms, and the right cerebellum is involved in phonological processing. Therefore, hyperactivation in the right cerebellum in readers with DD suggests that they may use it as a compensation for their deficient phonological processing, while hypoactivation in the left cerebellum may suggest reduced error monitoring in readers with DD. The finding of neurological alterations in the cerebellum is consistent with the previous findings of cerebellar deficit (Menghini et al., 2006; Yang et al., 2013) for which, some researchers argued that impaired articulatory motor control in the cerebellum leads to reading impairment (Nicolson et al., 2001; Stoodley and Stein, 2011). It also should be noticed that the cerebellum showed divergent patterns in structural studies and functional studies. Taken together, these results implicate the necessity of considering DD from a broader spectrum of developmental disorders.

It is still unclear why there is increased GMV but decreased activation in some brain regions. It may be due to the following reasons: (1) increased dendrites receiving more inhibitory input from other neurons; (2) abnormal neuronal migration deactivated the firing of neurons as a result of disrupted local microcircuits (Giraud and Ramus, 2013); (3) weaker input from other regions deactivated the target region and changed the structure of the region (Wang et al., 2019).

### The caudate

We also found decreased GMV and hyperactivation in readers with DD in the bilateral caudate in alphabetic languages. Previous studies have observed decreased GMV (Brown et al., 2001; Jagger-Rickels et al., 2018; McGrath and Stoodley, 2019; Tamboer et al., 2015) and hyperactivation in bilateral caudate in individuals with DD (Martin et al., 2016; Olulade et al., 2012; Pekkola et al., 2006; Richlan et al., 2010; Richlan et al., 2011; Rumsey et al., 1997). However, there are also studies that observed different patterns (Cheema et al., 2018), such as decreased activation in caudate (Perrachione et al., 2016). Furthermore, the GMV volume of the caudate in individuals with DD was found to be positively correlated with reading performance (Pernet et al., 2009; Tamboer et al., 2015), and the left caudate’s activation was correlated with longer reaction time in word reading only in individuals with DD (Cheema et al., 2018). The caudate plays an important role in procedural learning and phonological processing (Grahn et al., 2008; Tettamanti et al., 2005; Ullman et al., 2020). Decreased GMV and increased activation at the bilateral caudate might be caused by reduced dendrites and reduced inhibitory inputs received in individuals with DD (Achal et al., 2016; Finn et al., 2014). It may also be due to pre-existing local structural deficit leading to compensatory hyperactivation of the remaining part of the caudate. GMV reduction in basal ganglia was found in many other neuropsychiatric disorders, such as attention-deficit hyperactivity disorder (Frodl and Skokauskas, 2012; Mous et al., 2015; Nakao et al., 2011), autism spectrum disorder (Nickl-Jockschat et al., 2012) and major depression disorder (Husain et al., 1991; Lu et al., 2016). Altered myelination and neurotransmitters may contribute to the structural and functional alterations related to basal ganglia (Nord et al., 2019; Wichmann and DeLong, 2012).

### Conclusion

We found convergent functional and structural alterations in the left STG across different writing systems, suggesting a neural signature of DD, which might be associated with phonological deficit. We also found greater functional and structural alteration in the left dorsal IFG in morpho-syllabic languages than alphabetic languages, suggesting a language-specific effect of DD, which might be related to the special feature of whole-character-to-whole-syllable mapping in morpho-syllabic languages.

### Limitation

In this meta-analysis, we found convergent and divergent functional and structural alterations across writing systems. However, due to the limitations of voxel-based neuroimaging meta-analysis, the peak coordinate only provides limited information, therefore, future image-based meta-analysis studies should be conducted with full statistical images of the original studies (Muller et al., 2018).

## Materials and methods

### Literature retrieval and data extraction

**Figure 4.**
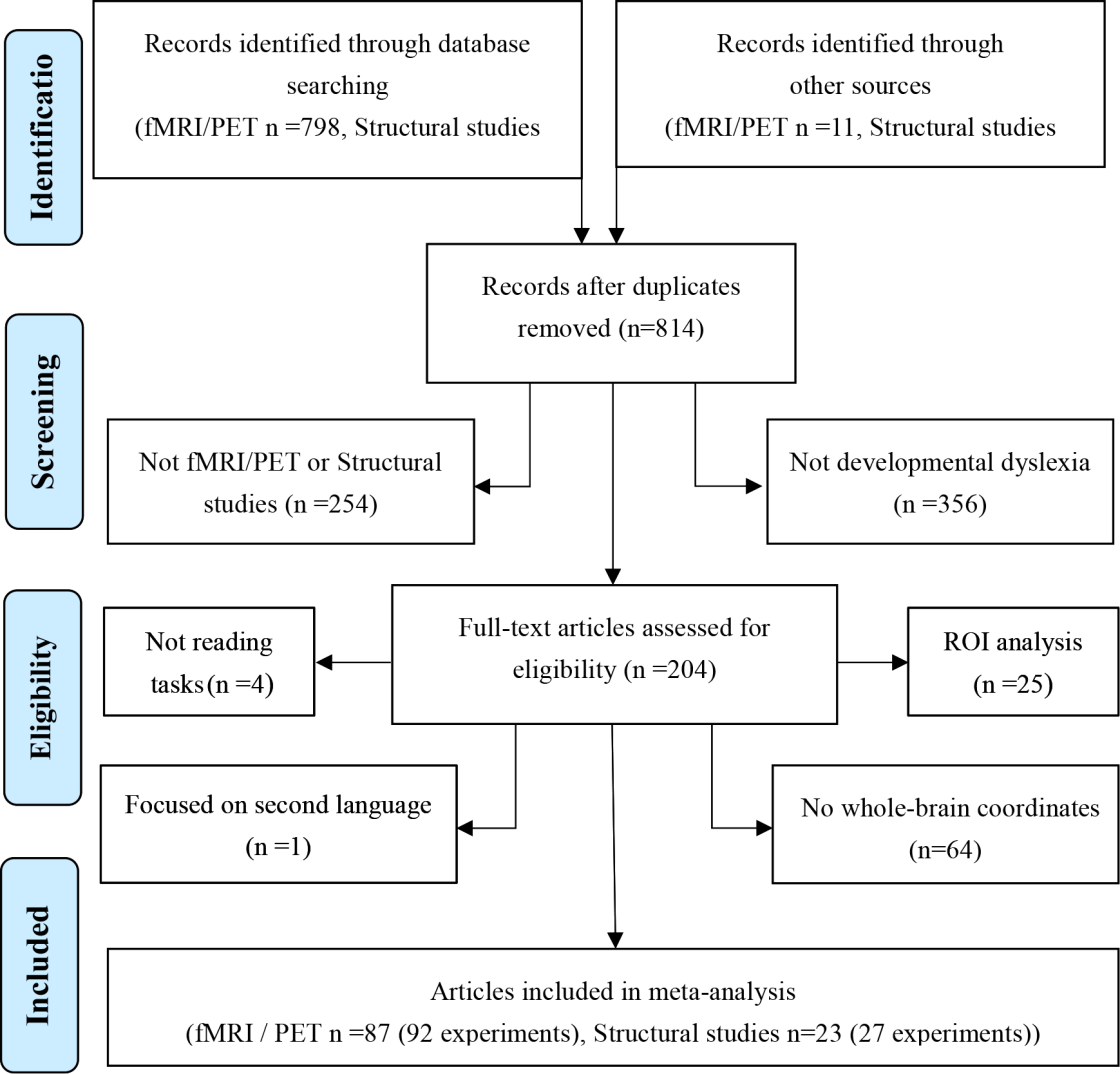
PRISMA flowchart of the selection process for included articles.

We searched in ‘PubMed’ (http://www.pubmed.org) and ‘Web of science’ for neuroimaging studies published from January 1986 to January 2020 using a combination of a condition term (i.e. dyslexia, reading disorder, reading impairment, reading difficulty or reading disability) and a technical term (i.e. fMRI, PET, voxel-based morphometry, VBM, or neuroimaging), for example, ‘dyslexia’ and ‘fMRI’. See the full list of key word combinations in the Supplementary file 2. Additionally, we manually added studies by checking the references of the selected papers that were missed in the search. The inclusion criteria were: (1) PET, fMRI, voxel-based morphometry (VBM) studies or structural studies using a volumetric FreeSurfer pipeline, (2) whole-brain results were reported, (3) direct group comparisons between readers with DD and age control readers were reported, (4) coordinates were reported in Talairach or MNI stereotactic space, and (5) studies on DD in the first language. The exclusion criteria were (1) studies with only ROI analysis, (2) resting-state studies, (3) studies that only included readers with DD or did not report group differences, (4) studies with direct group comparisons only between readers with DD and reading level control readers, (5) studies on children at risk for dyslexia, and (6) studies focused on non-linguistic tasks (Evans et al., 2014b; Margolis et al., 2020; Menghini et al., 2006; Yang et al., 2013). Finally, 119 experiments from 110 papers were included in this meta-analysis comprising 92 brain functional experiments (from 87 papers) and 27 brain structural experiments (from 23 papers) (see Table 9; Table 10 and Figure 4 for detail). From the original publications, we extracted peak coordinates, where there is a significant difference between controls and individuals with DD either in brain activation or regional GMV. We also extracted effect sizes and other information from the publications. In order to explore the language effect, we subdivided these studies into two groups according to the native language of the participants, namely, an alphabetic language group in which writing symbols represent phonemes, and a morpho-syllabic language group in which each writing symbol represents a morpheme with a syllable. This procedure resulted in 79 functional and 21 structural experiments for the alphabetic language group and 12 functional and six structural experiments for the morpho-syllabic language group.

**Table 9.** Functional studies included in the meta-analysis.

Studies	N(TD)	N(DD)	Age in months	Language	Writing system	Subject type	Tasks	Threshold	
								Voxel-wise	Cluster-wise
Bach et al., 2010	18	14	99.6	German	Alphabetic	Children	Covert reading task	p < 0.005	24 voxels*
Beneventi et al., 2009	13	11	160.4	Norwegian	Alphabetic	Children	Sequential verbal working memory task	p < 0.001	10 voxels
Beneventi et al., 2010a;	13	11	160.4	Norwegian	Alphabetic	Children	n-back task (Letter)	FDR p < 0.05	5 voxels
Beneventi et al., 2010b	14	12	160.3	Norwegian	Alphabetic	Children	n-back task (Picture)	FDR p < 0.05	
Blau et al., 2009	13	13	301.8	Dutch	Alphabetic	Adults	Letter–speech-sound integration task	p < 0.001	160 mm^3^*
Booth et al., 2007a	13	13	126.0	English	Alphabetic	Children	Word judgment task	p < 0.001	15 voxels
Boros et al., 2016	17	12	129.7	French	Alphabetic	Children	String detection and passive reading task	p < 0.001	FDR p < 0.05
Brambati et al., 2006	11	13	368.5	Italian	Alphabetic	Adults and adolescents	Word reading and pseudoword reading	p < 0.001	20 voxels
Brunswick et al., 1999	6	6	277.2	English	Alphabetic	Adults	Explicit reading task	p < 0.001	
Brunswick et al., 1999	6	6	294.0	English	Alphabetic	Adults	Implicit reading task	p < 0.001	
Cao et al., 2008	12	12	148.2	English	Alphabetic	Children	Visual word rhyming task	p < 0.001	10 voxels
Cao et al., 2017	13	17	134.0	Chinese	Morpho-syllabic	Children	Auditory rhyming task	p < 0.001	FDR p < 0.05
Cao et al., 2018	19	23	132.9	Chinese	Morpho-syllabic	Children	Visual spelling task	p < 0.001	FDR p < 0.05
Cao et al., 2020	17	16	137.3	Chinese	Morpho-syllabic	Children	Visual rhyming task	p < 0.001	FDR p < 0.05
Christodoulou et al., 2014	12	12	274.8	English	Alphabetic	Adults	Sentence reading	p < 0.001	FDR corrected
Chyl et al., 2019	24	24	105.4	Polish	Alphabetic	Children	Visual word reading	p < 0.001	50 voxels*
Conway et al., 2008	11	11	420.0	English	Alphabetic	Adults	Auditory working memory task	p < 0.005	150 mL
Cutting et al., 2013	19	20	147.02	English	Alphabetic	Adolescents	Lexical decision task	p < 0.005	34 voxels
Danelli et al., 2017	23	20	250.55	Italian	Alphabetic	Adults	Pseudoword reading, auditory letter-name rhyming task, visual motion stimulation task and motor sequence learning task	p < 0.001	FWE p < 0.05
Desroches et al., 2010	12	12	137.4	English	Alphabetic	Children	Auditory rhyming task	p < 0.001	15 voxels
Dufor et al., 2007	16	14	344.6	French	Alphabetic	Adult	Auditory phoneme categorization task	p < 0.01	
Eden et al., 2004	19	19	512.4	English	Alphabetic	Adults	Word repetition task and initial sound deletion task	p < 0.001	80 voxels
Farris et al., 2016	16	15	112.2	English	Alphabetic	Children	Object rhyming task	p < 0.001	10 voxels^**^
Feng et al., 2017	20	14	123.1	Chinese	Morpho-syllabic	Children	Character spelling task and character rhyming task	p < 0.001	12 voxels*
Francisco et al., 2018	20	21	303.7	Dutch	Alphabetic	Adults	1-back task	p < 0.001	FWE p < 0.05
Gaab et al., 2007	23	22	127.8	English	Alphabetic	Children	Sound discrimination task	p < 0.01	20 voxels
Georgiewa et al., 1999	17	17	168.0	German	Alphabetic	Children	Letter reading task, nonwords reading task, words reading task and phonological transformation task	p < 0.05	p < 0.05
Grande et al., 2011	25	20	115.1	German	Alphabetic	Children	Picture naming task and words reading task	p < 0.001	t10 voxels
Grunling et al., 2004	21	17	162.8	German	Alphabetic	Adolescents	Slash patterns matching task, letters matching task, words matching task, pseudoword matching task and pseudoword rhyming task	p < 0.01	10 voxels
Hancock et al., 2016	11	16	125.0	English	Alphabetic	Children	Word rhyming task	p < 0.01	50 voxels
Heim et al., 2010	20	16	113.9	German	Alphabetic	Children	First sound detection task, motion detection task, Posner attention task，auditory discrimination task	p < 0.001	p < 0.05
Heim et al., 2013	15	11	435.4	German	Alphabetic	Adults	Overt word reading	p < 0.05	
Heim et al., 2015	10	33	118.9	German	Alphabetic	Children	Overt word reading	FWE p < 0.05	100 voxels
Hernandez et al., 2013	16	15	252.7	French	Alphabetic	Adults	Word rhyming task and font matching task	p < 0.001*	
Higuchi et al., 2020	14	11	172.7	Japanese	Morpho-syllabic	Adolescents	Character/picture passive viewing task	p < 0.005	20 voxels
Hoeft et al., 2006	10	10	133.9	English	Alphabetic	Children	Visual word rhyming task	p < 0.001	10 voxels
Hoeft et al., 2007	19	19	172.8	English	Alphabetic	Adolescents	Visual word rhyming task	p < 0.001	10 voxels
Horowitz-Kraus et al., 2016	9	10	120.5	English	Alphabetic	Children	Narrative comprehension task	p < 0.001	FWE corrected
Hu et al., 2010	8	8	171.6	Chinese	Morpho-syllabic	Children	Sematic match task, word/ picture naming task	p < 0.001*	
Hu et al., 2010	10	11	164.5	English	Alphabetic	Children	Sematic match task, word/ picture naming task	p < 0.001*	
Ingvar et al., 2002	9	9	287.0	Swedish	Alphabetic	Adults	Word reading task and nonword reading task	p < 0.001	
Jaffe-Dax et al., 2018	19	20	302.2	Hebrew	Alphabetic	Adults	Tone frequency discrimination task		p < 0.05 corrected
Kast et al., 2011	13	12	314.5	German	Alphabetic	Adults	Lexical decision task	p < 0.001	30 voxels
Kovelman et al., 2012	12	12	108.4	English	Alphabetic	Children	Auditory words rhyming task and auditory words matching task	p < 0.001	25 voxels*
Kronbichler et al., 2006	15	13	187.9	German	Alphabetic	Adolescents	Sentence verification task	FDR p < 0.05	4 voxels
Kronschnabel et al., 2013	22	13	191.7	German	Alphabetic	Adolescents	Rapid serial visual stimulation detect task	p < 0.005	160 voxels*
Kronschnabel et al., 2014	22	13	190.9	German	Alphabetic	Adolescents	Target detection task	p < 0.005	160 voxels*
Landi et al., 2010	13	13	157.8	English	Alphabetic	Adolescents	Rhyming task and semantic categorization task	FDR p < 0.01	20 voxels
Langer et al., 2015	15	15	119.4	English	Alphabetic	Children	Sentence reading task	p < 0.005	50 voxels
Liu et al., 2012	11	11	142.8	Chinese	Morpho-syllabic	Children	Word rhyming task and semantic judgment task	p < 0.0002	18 voxels*
Liu et al., 2013a	14	14	141.8	Chinese	Morpho-syllabic	Children	Lexical match task and character rhyming task	p < 0.001	10 voxels
Lobier et al., 2014	12	12	129.6	French	Alphabetic	Adults	visual categorization of character task	p < 0.001	20 voxels
MacSweeney et al., 2009	7	7	343.5	English	Alphabetic	Adults	Picture rhyming task	p < 0.01	20 voxels
Maurer et al., 2011	16	11	136.6	German	Alphabetic	Children	Word matching task, pseudoword matching task, picture matching task	p < 0.01	30 voxels*
McCrory et al., 2000	6	8	275.0	English	Alphabetic	Adults	Words and pseudowords production	p < 0.001,	
McCrory et al., 2005	10	8	242.0	English	Alphabetic	Adults	Words reading and pictures naming	p < 0.05 corrected	
Meyler et al., 2008	12	23	129.6	English	Alphabetic	Children	Sentence comprehension	p < 0.002	10 voxels
Monzalvo et al., 2012	23	23	130.0	French	Alphabetic	Children	Passive picture/word viewing task and passive sentence listening task	p < 0.001	p < 0.05 corrected
Olulade et al., 2012	9	6	247.7	English	Alphabetic	Adults	Word rhyme task3-D spatial rotations	p < 0.005	10 voxels
Olulade et al., 2015	12	16	120.5	English	Alphabetic	Children	Implicit word reading	p < 0.001	20 voxels
Paulesu et al., 2001	36	36	286.4	English, French, Italian	Alphabetic	Adults	Word and non-word reading task	p< 0.001	corrected
Paulesu et al., 1996	5	5	314.5	English	Alphabetic	Adults	Letter rhyming and letter memory	p < 0.001	
Pecini et al., 2011	13	13	276.0	Italian	Alphabetic	Adults and adolescents	Rhyme-generation task	p < 0.05 corrected	100 mm^3^
Pekkola et al., 2006	10	10	330.6	Finnish	Alphabetic	Adults	Audio-visual speech perception	z > 1.8	p < 0.05 corrected
Perrachione et al., 2016	19	19	279.6	English	Alphabetic	Adults	Speech perception	p < 0.001	p < 0.001 FDR
Perrachione et al., 2016	24	23	267.0	English	Alphabetic	Adults	Spoken words listening, Written words, objects, and faces viewing	p < 0.001	p < 0.001 FDR
Perrachione et al., 2016	25	26	95.4	English	Alphabetic	Children	Spoken words listening, Written words, objects, and faces viewing	p < 0.001	p < 0.05 FDR
Peyrin et al., 2011	12	12	120.0	French	Alphabetic	Children	Categorical matching task	p < 0.001	15 voxels
Prasad et al., 2020	15	16	144.0	Hindi	Syllabic	Children and adolescents	Auditory rhyming task, picture-naming task and semantic tasks	p < 0.001	
Reilhac et al., 2013	12	12	306.6	French	Alphabetic	Adults	Perceptual matching task	p < 0.001	15 voxels
Richlan et al., 2010	18	15	215.8	German	Alphabetic	Adults and adolescents	phonological decision task	p < 0.005	20 voxels
Rimrodt et al., 2009	15	14	141.0	English	Alphabetic	Children	Sentence comprehension task	p < 0.001	78 voxels*
Ruff et al., 2002	11	6	348.7	French	Alphabetic	Aadults	Passive listening task	p < 0.01	38 voxels
Rumsey et al., 1997	14	17	313.2	English	Alphabetic	Adults	Pronunciation task and lexical decision task	p < 0.01	
Schulz et al., 2008	21	12	137.7	German	Alphabetic	Children	Sentence reading task	p < 0.001	
Schulz et al., 2009	15	15	138.0	German	Alphabetic	Children	Sentence reading task	p < 0.001	
Siok et al., 2004	8	8	132.0	Chinese	Morpho-syllabic	Children	Homophone judgement task and lexical decision task	p < 0.001	20 voxels
Siok et al., 2008	12	12	131.5	Chinese	Morpho-syllabic	Children	Character rhyming task	p < 0.005	10 voxels
Siok et al., 2009	12	12	131.5	Chinese	Morpho-syllabic	Children	Font size judgment task	p < 0.05 FDR corrected	10 voxels
Steinbrink et al., 2012	12	14	223.7	German	Alphabetic	Adults and adolescents	Syllable discrimination	p < 0.05 FWE corrected	
Temple et al., 2000	10	8	362.7	English	Alphabetic	Adults	Pitch discrimination task	p < 0.001	
Temple et al., 2001	15	24	127.5	English	Alphabetic	Children	Letter rhyming task, letter matching task	p < 0.001	20 voxels
van der Mark et al., 2009	24	18	136.1	German	Alphabetic	Children	Phonological lexical decision task	p < 0.001	10 voxels
van Ermingen-Marbach et al., 2013a	13	17	117.2	German	Alphabetic	Children	Phoneme detection task	p < 0.001	10 voxels
van Ermingen-Marbach et al., 2013a	13	14	116.4	German	Alphabetic	Children	Phoneme detection task	p < 0.001	10 voxels
van Ermingen-Marbach et al., 2013b	10	32	117.0	German	Alphabetic	Children	Initial phoneme deletion task	p < 0.01	30 voxels
Vasic et al., 2008	13	12	219.6	German	Alphabetic	Adults	Verbal working memory task	p < 0.005	p < 0.05
Waldie et al., 2013	16	12	365.1	English	Alphabetic	Adults	Go/no-go lexical decision task	p < 0.001	
Weiss et al., 2016	22	21	325.1	Hebrew	Alphabetic	Adults	Word reading task	p < 0.001	50 voxels
Wimmer et al., 2010	19	20	247.6	German	Alphabetic	Adults and adolescents	Phonological lexical decision task	p < 0.005	10 voxels
Yang and Tan, 2020	16	16	123.5	Chinese	Morpho-syllabic	Children	Homophone judgments task and component judgments task	p < 0.005	25 voxels*
Zuk et al., 2018	13	11	114.0	English	Alphabetic	Children	First sound matching task	p < 0.005	50 voxels

* equivalent to p < 0.05 corrected; ** equivalent to p < 0.01 corrected.

**Table 10.** Structural studies included in the meta-analysis.

Study	N(TD)	N(DD)	Age in months	Language	Writing system	Subject type	Threshold	
							Voxel-wise	Cluster-wise
Adrian-Ventura et al., 2020	12	13	146.2	Spanish	Alphabetic	Children	p < 0.05 FWE corrected	750 voxels
Brambati et al., 2004	11	10	352.8	Italian	Alphabetic	Adults and adolescents	p < 0.05 corrected for small brain volume	
Brown et al., 2001	14	16	288.0	English	Alphabetic	Adults	p < 0.05	p < 0.05
Eckert et al., 2005	13	13	136.5	English	Alphabetic	Children	p < 0.001	
Evans et al., 2014a	14	14	505.8	English	Alphabetic	Adults	*P*p< 0.001	p < 0.05
Evans et al., 2014a	13	13	371.4	English	Alphabetic	Adults	p < 0.001	p< 0.05
Evans et al., 2014a	15	15	107.4	English	Alphabetic	Children	p < 0.001	p < 0.05
Evans et al., 2014a	17	17	115.2	English	Alphabetic	Children	p < 0.001	p < 0.05
Hoeft et al., 2007	19	19	172.8	English	Alphabetic	Children and adolescents	p < 0.01	p < 0.01
Jagger-Rickels et al., 2018	32	17	114.2	English	Alphabetic	Children	p < 0.001	
Jednorog et al., 2014	35	46	123.5	Polish	Alphabetic	Children	p < 0.001	p < 0.05
Jednorog et al., 2015	106	130	123.9	French, German, Polish	Alphabetic	Children	p < 0.001	150 voxels*
Krafnick et al., 2014	15	15	118.2	English	Alphabetic	Children	*P*p< 0.01	p < 0.01 FWE corrected
Kronbichler et al., 2008	15	13	187.9	German	Alphabetic	Adolescents	p < 0.005	
Liu et al., 2013b	18	18	141.4	Chinese	Morpho-syllabic	Cchildren	p < 0.001	196 voxels
Menghini et al., 2008	10	10	489.0	Italian	Alphabetic	Adult	p < 0.005	p < 0.05 corrected
Moreau et al., 2019	12	12	352.2	English	Alphabetic	Adult	p < 0.05 FWE corrected	
Pernet et al., 2009	39	38	336.0	French	Alphabetic	Adult	p< 0.05 FDR corrected	
Silani et al., 2005	32	32	304.5	Italian, French, English	Alphabetic	Adults		
Siok et al., 2008	16	16	132.0	Chinese	Morpho-syllabic	Children	p < 0.05 FWE corrected	50 voxels
Steinbrink et al., 2008	8	8	262.8	German	Alphabetic	Adults	p < 0.05 FDR corrected	
Tamboer et al., 2015	57	37	245.3	Dutch	Alphabetic	Adults	p < 0.05	200 voxels
Vinckenbosch et al., 2005	10	13	282.0	French	Alphabetic	Adults	p < 0.01	p < 0.05
Wang et al., 2019	17	27	134.0	Chinese	Morpho-syllabic	Children	p < 0.001	p < 0.05 FWE corrected
Xia et al., 2016	12	12	132.0	Chinese	Morpho-syllabic	Children	p < 0.001	
Xia et al., 2016	12	12	169.2	Chinese	Morpho-syllabic	Children	p < 0.001	
Yang et al., 2016	14	9	149.0	Chinese	Morpho-syllabic	Children	p < 0.001	111 voxels*

* equivalent to 0.05 corrected; ** equivalent to 0.01 corrected.

### Voxel-wise meta-analysis

After data acquisition, we conducted a voxel-wise meta-analysis using the anisotropic effect-size version of Signed Differential Mapping software (AES-SDM version 5.14, see http://www.sdmproject.com) separately for functional studies and structural studies in alphabetic languages and morpho-syllabic languages. Unlike other coordinate-based meta-analysis methods such as Activation likelihood estimation (ALE) or Multilevel peak Kernel density analysis (MKDA), AES-SDM combined the peak coordinates with the statistical parameter maps to increase the sensitivity of the analysis (Radua et al., 2012a). Data were first preprocessed with the statistical parameter maps and the peak coordinates were convolved with a fully anisotropy un-normalized Gaussian kernel (ɑ = 1) (full width at half maximum = 20 mm) to recreate the effect size map and the corresponding variance map for each study (Radua et al., 2012a; Radua et al., 2014). Then, a random-effect model was set up to calculate the differences between the DD group and the control group. Five hundred permutations were performed to ensure the stability of the analysis. Finally, the results of the standard meta-analysis were thresholded at peak height of the mean effect size SDM-Z = 1, uncorrected p = 0.005 at the voxel level and 150 voxels at the cluster level, which is stricter than the threshold suggested by Radua et al., 2012a (peak height SDM-Z = 1, uncorrected p = 0.005 at the voxel level and 10 voxels at the cluster level) in order to avoid false-positive results and gain enough sensitivity.

In order to identify differences between the two language groups, we conducted a direct comparison between the alphabetic language group and the morpho-syllabic language group for functional studies and structural studies separately, using SDM linear model function. The threshold was set at peak height SDM-Z = 1, voxel level uncorrected p = 0.005 and 150 voxels at the cluster level.

To find out the common language difference between the structural and functional studies, we conducted a conjunction minimum analysis (Friston et al., 1999; Nichols et al., 2005) using the image calculation function of SPM12 (https://www.fil.ion.ucl.ac.uk) between the language differences in the structural studies and the language differences in the functional studies.

To test the stability of the meta-analysis results, we conducted a whole-brain jack-knife sensitivity analysis. The standard meta-analysis was repeated n times (n = 79 for functional experiments in alphabetic languages, n = 12 for functional experiments in morpho-syllabic languages, n = 21 for structural experiments in alphabetic languages, n = 6 for structural experiments in morpho-syllabic languages) but leaving out one experiments each time, to determine whether the results remained significant.

### Multimodal meta-analysis

Because we were interested in the convergence between functional deficits and structural deficits, a multimodal meta-analysis was conducted in alphabetic languages and morpho-syllabic languages separately, which provided an efficient way to combine two meta-analyses in different modalities. The union probabilities of the meta-analytical maps of functional studies and structural studies were estimated and then thresholded at the peak height p = 0.00025, with a voxel level uncorrected p = 0.0025 and 150 voxels at the cluster level, which was stricter than the one suggested by Radua et al., 2012b; Radua et al., 2013 (peak height p = 0.00025, with a voxel level uncorrected p = 0.0025 and 10 voxels at cluster level).

To find out the common multimodal deficits in the two language groups, we conducted a conjunction minimum analysis (Friston et al., 1999; Nichols et al., 2005) using the image calculation function of SPM12 (https://www.fil.ion.ucl.ac.uk) between the multimodal deficits in the alphabetic group and the morpho-syllabic group.

### Confirmation study

Because there were studies on both adults and children in the alphabetic group, whereas most of the morpho-syllabic studies were on children, the language difference may be due to the unmatched age range in the two groups of studies. In order to eliminate the influence of the confound, we conducted a confirmation analysis with only studies on children in the alphabetic group (n = 36 for the functional studies and n = 8 for the structure studies). Then, we compared the alphabetic and morpho-syllabic groups for functional studies and structural studies separately.

We conducted another confirmation analysis with 10 English studies (Booth et al., 2007a; Cao et al., 2008; Farris et al., 2016; Hancock et al., 2016; Hu et al., 2010; Langer et al., 2015; Meyler et al., 2008; Olulade et al., 2015; Rimrodt et al., 2009; Temple et al., 2001) and 10 Chinese studies (Cao et al., 2018; Cao et al., 2020; Feng et al., 2017; Hu et al., 2010; Liu et al., 2012; Liu et al., 2013a; Siok et al., 2004; Siok et al., 2008; Siok et al., 2009; Yang and Tan, 2020), because different languages were included in the alphabetic language group and orthographic depth makes a difference as found in previous research (Martin et al., 2016). Therefore, we only included English studies in the alphabetic group in this confirmation analysis. The two subgroups were also matched on participants’ age (mean age = 10.95 years for English studies, mean age = 11.40 years for Chinese studies), number of studies and task (visual word tasks). Then, we conducted a direct comparison between the Chinese studies and English studies. The threshold was set at peak height SDM-Z = 1 and voxel level uncorrected p = 0.005 with 150 voxels at the cluster level.

## Data Availability

All data generated or analysed during this study are included in the manuscript and supporting files. Meta-analysis data is deposited to Dryad. The following dataset was generated: YanX 2021meta-analysis dataDryad Digital Repository10.5061/dryad.0p2ngf222

## Additional files

Supplementary file 1. Additional results for the meta-analysis. (a) Functional deficits in children with DD in alphabetic languages (b) Structural deficits in children with DD in alphabetic languages (c) Direct comparations between functional studies in alphabetic languages and morpho-syllabic languages for children with DD (d) Direct comparations between structural studies in alphabetic languages and morpho-syllabic languages for children with DD (e) Language differences in functional studies between the two well-matched groups in the confirmation analysis (f) Abnormal brain functions found in the current study that were reported in each functional study of the alphabetic language group (g) Abnormal brain structures found in the current study that were reported in each structural study of the alphabetic group (h) Abnormal brain functions found in the current study that were reported in each functional study of the morpho-syllabic group (i) Abnormal brain structures found in the current study that were reported in each structural study of the morpho-syllabic group.

Supplementary file 2. Key words used in literature retrieval.

Transparent reporting form
