# Supplementary material for: Convergent and divergent brain structural and functional abnormalities associated with developmental dyslexia
Source: eLife. 2021 Sep 27;10:e69523. doi: 10.7554/eLife.69523 (PMC8497057; doi:10.7554/eLife.69523)
Supplement: Supplementary file 1. — (a) Functional deficits in children with DD in alphabetic languages (b) Structural deficits in children with DD in alphabetic languages (c) Direct comparations between functional studies in alphabetic languages and morpho-syllabic languages for children with DD (d) Direct comparations between structural studies in alphabetic languages and morpho-syllabic languages for children with DD (e) Language differences in functional studies between the two well-matched groups in the confirmation analysis (f) Abnormal brain functions found in the current study that were reported in each functional study of the alphabetic language group (g) Abnormal brain structures found in the current study that were reported in each structural study of the alphabetic group (h) Abnormal brain functions found in the current study that were reported in each functional study of the morpho-syllabic group (i) Abnormal brain structures found in the current study that were reported in each structural study of the morpho-syllabic group. [file elife-69523-supp1.docx]

**Table 1a.** Functional deficits in children with DD in alphabetic languages

| Regions | | MNI coordinate | SDM-Z | | P | Voxels | Cluster breakdown (Voxels) |
| --- | --- | --- | --- | --- | --- | --- | --- |
|  | *Hypoactivation in DD* | | | | | | |
| Left IPL | | -46,-42,46 | 3.348 | | 0.0000 | 4018 | Left IPL, BA 40 (842)  Left MTG, BA 37 (396)  Left fusiform gyrus, BA 37 (305)  Left ITG, BA 37 (294)  Left MTG, BA 21 (263)  Left ITG, BA 20 (197)  Left angular gyrus, BA 39 (182) |
|  | |  |  | |  |  |  |
|  | |  |  | |  |  |  |
|  | |  |  | |  |  |  |
|  | |  |  | |  |  |  |
|  | |  |  | |  |  |  |
|  | |  |  | |  |  |  |
| Right precentral gyrus | | 44,4,46 | 2.317 | | 0.0001 | 352 | Right precentral gyrus, BA 6 (212) |
| Left IFG, opercular part | | -48,10,4 | 2.287 | | 0.0002 | 230 |  |
| *Hyperactivation in DD* | |  |  |  | | |  |
| Right Cerebellum | | 22,-58,-28 | -1.283 | | 0.0002 | 810 | Right cerebellum, lobule VI, BA 37 (229) |

**Table 1b.** Structural deficits in children with DD in alphabetic languages

| Regions | | MNI coordinate | SDM-Z | | P | Voxels | Cluster breakdown (Voxels) |
| --- | --- | --- | --- | --- | --- | --- | --- |
|  | *Decreased GMV in DD* | | | | | | |
| Left thalamus | | -6,-20,8 | 2.429 | | 0.0001 | 420 | Left thalamus (167) |
| Right precentral gyrus | | 40,-16,42 | 1.620 | | 0.0019 | 274 |  |
| Left inferior occipital gyrus | | -12,-96,-4 | 1.498 | | 0.0026 | 187 |  |
| *Increased GMV in DD* | |  |  |  | | |  |
| Left IPL | | -38,-38,36 | -2.423 | | 0.0000 | 1167 | Left IPL, BA 40 (459)  Left IPL, BA 2 (158) |

**Table 1c.** Direct comparations between functional studies in alphabetic languages and morpho-syllabic languages for children with DD

| Regions | MNI coordinate | SDM-Z | P | Voxels | Cluster breakdown (Voxels) |
| --- | --- | --- | --- | --- | --- |
| *Hypoactivation in DD*  *Alphabetic languages > Morpho-syllabic languages* | | | | | |
| Right ITG | 50,-20,10 | 1.208 | 0.0000 | 1590 | Right STG, BA 22 (333)  Right rolandic operculum, BA 48 (249)  Corpus callosum (217)  Right STG, BA 48 (159) |
| Left STG | -48,-2,-8 | 1.133 | 0.0001 | 568 | Left STG, BA 48 (156) |
| Left superior occipital gyrus | -26,-88,24 | 1.032 | 0.0003 | 391 |  |
| Left MTG | -50,-66,8 | 1.040 | 0.0002 | 154 |  |
| *Morpho-syllabic languages > Alphabetic languages* | | | | | |
| Left IFG, opercular part | -48,8,28 | -4.366 | 0.0000 | 2215 | Left precentral gyrus, BA 6 (541)  Left IFG, opercular part, BA 44 (286)  Left precentral gyrus, BA 44 (191)  Left IFG, triangular part, BA 48 (183)  Corpus callosum (173) |
| *Hyperactivation in DD*  *Alphabetic languages > Morpho-syllabic languages* | | | | | |
| - | - | - | - | - |  |
| *Morpho-syllabic languages > Alphabetic languages* | | | | | |
| Right precentral gyrus | 40,-20,54 | -2.522 | 0.0000 | 1566 | Right precentral gyrus, BA 6 (550)  Right precentral gyrus, BA 4 (321)  Right postcentral gyrus, BA 3 (287)  Right postcentral gyrus, BA 3 (142) |

**Table 1d.** Direct comparations between structural studies in alphabetic languages and morpho-syllabic languages for children with DD

| Regions | MNI coordinate | SDM-Z | P | Voxels | Cluster breakdown (Voxels) |
| --- | --- | --- | --- | --- | --- |
| *Decreased GMV in DD*  *Alphabetic languages > Morpho-syllabic languages* | | | | | |
| Left thalamus | -6,-20,4 | 1.346 | 0.0001 | 448 | Left thalamus (180) |
| Right precentral gyrus | 40,-16,42 | 1.046 | 0.0005 | 463 |  |
| *Morpho-syllabic languages > Alphabetic languages* | | | | | |
| Left STG | -48,12,-20 | -2.769 | 0.0000 | 1250 | Left STG, BA 38 (347) |
| Left STG | -56,-42,22 | -2.489 | 0.0001 | 585 | Left STG, BA 42 (150) |
| Left MFG | -32,22,44 | -2.423 | 0.0001 | 358 |  |
| Left IFG, opercular part | -52,10,24 | -1.992 | 0.0008 | 278 |  |
| Left superior occipital gyrus | -20,-70,16 | -2.470 | 0.0001 | 259 |  |
| Left insula | -32,14,8 | -1.869 | 0.0014 | 174 |  |
| *Increased GMV in DD*  *Alphabetic languages > Morpho-syllabic languages* | | | | | |
| Left IPL | -42,-36,36 | 1.436 | 0.000 | 1062 | Left IPL, BA 40 (419) |
| *Morpho-syllabic languages > Alphabetic languages* | | | | | |
| Right STG | 34,6,-26 | 1.650 | 0.0001 | 1693 | Right STG, BA 38 (266) |

**Table 1e.** Language differences in functional studies between the two well-matched groups in the confirmation analysis

| Regions | MNI coordinate | SDM-Z | P | Voxels | Cluster breakdown (Voxels) |
| --- | --- | --- | --- | --- | --- |
| *Hypoactivation in DD*  *Alphabetic languages > Morpho-syllabic languages* | | | | | |
| Left ITG | -60,-38,4 | 1.665 | 0.0001 | 1044 | left ITG, BA 20 (569)  left MTG, BA 20 (185) |
| Left MOG | -20,-88,-20 | 1.785 | 0.0001 | 606 | Corpus callosum (222) |
| Left MOG | -48,-68,4 | 1.489 | 0.0005 | 200 |  |
| *Morpho-syllabic languages > Alphabetic languages* | | | | | |
| Left precentral gyrus | -56,4,26 | -2.865 | 0.0000 | 2311 | Left precentral gyrus, BA 6 (592)  Left IFG, opercular part, BA 44 (286)  Left precentral gyrus, BA 44 (182)  Corpus callosum (173) |
| *Hyperactivation in DD*  *Alphabetic languages > Morpho-syllabic languages* | | | | | |
| Right supramarginal gyrus | 60,-48,26 | -1.133 | 0.0004 | 715 |  |
| Right hippocampus | 26,-10,-12 | -1.304 | 0.0001 | 585 |  |
| Right supplementary motor area | 4,8,48 | -1.105 | 0.0005 | 390 |  |
| *Morpho-syllabic languages > Alphabetic languages* | | | | | |
| Right precentral gyrus | 40,-20,54 | -1.536 | 0.0001 | 792 | Right precentral gyrus, BA 6 (236)  Right precentral gyrus, BA 4 (208)  Right postcentral gyrus, BA 3 (180) |

**Table 1f.** Abnormal brain functions found in the current study that were reported in each functional study of the alphabetic language group

|  | left OT | Left TP | left IFG/MFG | Right MOG | Right STG | Right cerebellum | Left caudate nucleus | Right caudate nucleus |
| --- | --- | --- | --- | --- | --- | --- | --- | --- |
| (Bach et al., 2010) |  | † | *† |  | † |  |  |  |
| (Beneventi et al., 2009) |  |  |  |  |  |  |  |  |
| (Beneventi et al., 2010a) |  | * |  |  |  |  |  |  |
| (Beneventi et al., 2010b) |  |  | * |  |  | * |  |  |
| (Blau et al., 2009) |  | * |  |  | * |  |  |  |
| (Booth et al., 2007a) |  |  | * |  |  |  |  |  |
| (Boros et al., 2016) | * | * | * | * |  |  |  |  |
| (Brambati et al., 2006) | * | * | * |  |  | * |  |  |
| (Brunswick et al., 1999) | * |  |  |  |  |  | * |  |
| (Brunswick et al., 1999) | * | * |  |  |  |  |  |  |
| (Cao et al., 2008) |  | * | * |  |  |  |  |  |
| (Christodoulou et al., 2014) |  | * | * |  |  |  |  |  |
| (Chyl et al., 2018) | * |  | * |  |  |  |  |  |
| (Conway et al., 2008) |  | *† |  |  |  |  |  |  |
| (Cutting et al., 2013) | * |  | * |  |  |  |  |  |
| (Danelli et al., 2017) | * |  |  |  |  |  |  |  |
| (Desroches et al., 2010) | * |  |  |  |  |  |  |  |
| (Dufor et al., 2007) |  | * |  |  | * | * |  |  |
| (Eden et al., 2004) |  | * |  |  |  |  |  |  |
| (Farris et al., 2016) | * |  |  | * |  |  |  |  |
| (Francisco et al., 2018) |  |  |  |  |  |  |  |  |
| (Gaab et al., 2007) |  |  | * |  |  |  |  |  |
| (Georgiewa et al., 1999) |  | † | * |  |  |  |  |  |
| (Grande et al., 2011) |  | * | † |  |  |  |  |  |
| (Grunling et al., 2004) |  | † | † |  |  |  |  |  |
| (Hancock et al., 2016) |  | * | * |  |  |  |  |  |
| (Heim et al., 2010) |  |  | * |  | * |  |  |  |
| (Heim et al., 2013) |  | *† | *† |  |  |  |  |  |
| (Heim et al., 2015) |  |  |  | * | * |  |  |  |
| (Hernandez et al., 2013) |  |  |  |  |  | † |  |  |
| (Hoeft et al., 2006) |  | * | * |  |  |  |  |  |
| (Hoeft et al., 2007) | * | * | † |  | * |  | † |  |
| (Horowitz-Kraus et al., 2016) |  |  |  |  | * |  |  |  |
| (Hu et al., 2010) | * | * | * |  |  |  |  |  |
| (Ingvar et al., 2002) |  |  |  |  |  |  |  |  |
| (Jaffe-Dax et al., 2018) |  | * |  |  |  |  |  |  |
| (Kast et al., 2011) |  | * |  | * |  |  |  |  |
| (Kovelman et al., 2012) |  |  |  |  |  |  |  |  |
| (Kronbichler et al., 2006) |  | * | † |  |  |  |  | † |
| (Kronschnabel et al., 2013) | * | † | * | * | † |  |  |  |
| (Kronschnabel et al., 2014) |  |  | † |  |  | † |  |  |
| (Landi et al., 2010) | * |  | * |  |  |  | * |  |
| (Langer et al., 2015) | * | * |  |  |  | * |  |  |
| (Lobier et al., 2014) | * |  | † |  |  |  |  |  |
| (MacSweeney et al., 2009) |  |  | † |  |  |  |  |  |
| (Maurer et al., 2011) | * | * |  | * |  | * |  |  |
| (McCrory et al., 2000) |  |  |  |  | * |  |  |  |
| (McCrory et al., 2005a) | * |  |  |  |  |  |  |  |
| (Meyler et al., 2008) |  | * | * |  |  |  |  |  |
| (Monzalvo et al., 2012) |  |  |  |  |  |  |  |  |
| (Olulade et al., 2012) | * | * | * | † |  |  |  | † |
| (Olulade et al., 2015) | * |  |  | * |  |  |  |  |
| (Paulesu et al., 2001) | * | * |  |  |  |  |  |  |
| (Paulesu et al., 1996) |  | * |  |  |  |  |  |  |
| (Pecini et al., 2011) |  | * | * |  |  |  |  |  |
| (Pekkola et al., 2006) | † | † |  |  |  |  | † | † |
| (Perrachione et al., 2016) |  | * |  |  | * |  |  |  |
| (Perrachione et al., 2016) |  | * |  | * | * |  |  |  |
| (Perrachione et al., 2016) |  | * |  |  |  |  |  |  |
| (Peyrin et al., 2011) |  |  |  |  |  |  |  |  |
| (Prasad et al., 2020) |  |  |  |  |  | † |  |  |
| (Reilhac et al., 2013) | * | * | * | * |  | * |  |  |
| (Richlan et al., 2010) | * | * | * |  |  | † | † | † |
| (Rimrodt et al., 2009) |  | † |  |  |  |  |  |  |
| (Ruff et al., 2002) |  | * | *† |  |  |  |  |  |
| (Rumsey et al., 1997) | * | * |  |  |  | † |  | † |
| (Schulz et al., 2008) |  | * |  |  |  |  |  |  |
| (Schulz et al., 2009) | * | * |  |  |  |  |  |  |
| (Steinbrink et al., 2012) |  |  | * |  |  |  |  |  |
| (Temple et al., 2000) |  |  | * |  |  | * |  |  |
| (Temple et al., 2001) |  |  | † | * |  |  |  |  |
| (van der Mark et al., 2009) | * | * | * |  |  |  |  |  |
| (van Ermingen-Marbach et al., 2013a) |  |  | † |  |  | † |  |  |
| (van Ermingen-Marbach et al., 2013a) |  |  | † | † |  | † |  |  |
| (van Ermingen-Marbach et al., 2013b) |  |  | *† |  |  |  |  |  |
| (Vasic et al., 2008) |  | † | *† |  |  |  |  |  |
| (Waldie et al., 2013) |  |  | † | * |  |  |  |  |
| (Weiss et al., 2016) |  | * | * | * |  |  |  |  |
| (Wimmer et al., 2010) | * | * | *† |  |  |  | † | † |
| (Zuk et al., 2018) |  | * |  |  |  |  |  |  |
| Total number | 26 (1)/79 | 39 (9)/79 | 31 (17)/79 | 12 (2)/79 | 9 (2)/79 | 7 (7)/79 | 2 (4)/ 79 | 0 (6)/79 |

*, TD>DD; †, DD>TD; Numbers in the last line, the first number is the number of studies that showed decreased brain activation in DD, the number in the brackets is the number of studies that showed increased activation in DD, the last number is the total number of experiments included in the analysis.

**Table 1g.** Abnormal brain structures found in the current study that were reported in each structural study of the alphabetic group

|  | Left ventral IFG | Left STG | Right STG/MTG | Right caudate | Left IPL |
| --- | --- | --- | --- | --- | --- |
| (Adrian-Ventura et al., 2020) | * |  |  |  |  |
| (Brambati et al., 2004) |  | * | * |  |  |
| (Brown et al., 2001) | * | * |  | * |  |
| (Eckert et al., 2005) | * |  |  |  |  |
| (Evans et al., 2014b) |  |  |  |  |  |
| (Evans et al., 2014b) |  |  |  |  |  |
| (Evans et al., 2014b) |  |  |  |  | * |
| (Evans et al., 2014b) |  |  |  |  |  |
| (Hoeft et al., 2007) |  | * | * |  | * |
| (Jagger-Rickels et al., 2018) |  |  |  | * |  |
| (Jednorog et al., 2014) | * |  |  |  |  |
| (Jednorog et al., 2015) |  |  |  |  | † |
| (Krafnick et al., 2014) |  |  | * |  |  |
| (Kronbichler et al., 2008) |  | † |  |  |  |
| (Menghini et al., 2008) |  |  |  |  |  |
| (Moreau et al., 2019) |  |  |  |  |  |
| (Pernet et al., 2009) |  |  |  |  |  |
| (Silani et al., 2005) |  |  |  |  |  |
| (Steinbrink et al., 2008) |  | * | * |  |  |
| (Tamboer et al., 2015) |  |  | † | * | † |
| (Vinckenbosch et al., 2005) |  |  |  |  |  |
| Total number | 4 (0)/21 | 4 (1)/21 | 4 (1)/21 | 3 (0)/21 | 2 (2)/21 |

*, TD>DD; †, DD>TD; Numbers in the last line, the first number is the number of studies that showed decreased GMV in DD, the number in the brackets is the number of studies that showed increased GMV in DD, the last number is the total number of experiments included in the analysis.

**Table 1h.** Abnormal brain functions found in the current study that were reported in each functional study of the morpho-syllabic group

|  | Left dorsal IFG | Left supramarginal gyrus/IPL | Left ITG/Fusiform gyrus | Right precentral gyrus | Right MTG/STG |
| --- | --- | --- | --- | --- | --- |
| (Cao et al., 2017) | * | * |  | † |  |
| (Cao et al., 2018) | * |  | * | † |  |
| (Cao et al., 2020) | * | * |  |  |  |
| (Feng et al., 2017) | |  |  |  |  |
| (Higuchi et al., 2020) | |  |  |  |  |
| (Hu et al., 2010) | * |  | * |  |  |
| (Liu et al., 2012) | * |  |  |  |  |
| (Liu et al., 2013b) | * |  |  |  |  |
| (Siok et al., 2004) | * |  |  | † |  |
| (Siok et al., 2008) | * |  |  |  |  |
| (Siok et al., 2009) |  | * |  |  |  |
| (Yang & Tan, 2020) | * |  |  |  | † |
| Total number | 9 (0)/12 | 3 (0)/12 | 2 (0)/12 | 0 (3)/12 | 0 (1)/12 |

*, TD>DD; †, DD>TD; Numbers in the last line, the first number is the number of studies that showed decreased brain activation in DD, the number in the brackets is the number of studies that showed increased activation in DD, the last number is the total number of experiments included in the analysis.

**Table 1i.** Abnormal brain structures found in the current study that were reported in each structural study of the morpho-syllabic group

|  | Left STG/MTG | Left calcarine cortex | Left MFG/dorsal IFG | Right STG/MTG | Right precuneus |
| --- | --- | --- | --- | --- | --- |
| (Liu et al., 2013a) | * |  | * |  |  |
| (Siok et al., 2008) | * |  | * |  |  |
| (Wang et al., 2019) | * |  |  | † |  |
| (Xia et al., 2016) | * | * | * |  |  |
| (Xia et al., 2016) | * | * | * |  |  |
| (Yang et al., 2016) | * |  | * | † | † |
| Total number | 6 (0)/6 | 2 (0)/6 | 5 (0)/6 | 0 (2)/6 | 0 (1)/6 |

*, TD>DD; †, DD>TD; Numbers in the last line, the first number is the number of studies that showed decreased GMV in DD, the number in the brackets is the number of studies that showed increased GMV in DD, the last number is the total number of experiments included in the analysis.
