# Supplementary material for: Convergent and divergent brain structural and functional abnormalities associated with developmental dyslexia
Source: eLife. 2021 Sep 27;10:e69523. doi: 10.7554/eLife.69523 (PMC8497057; doi:10.7554/eLife.69523)
Supplement: Supplementary file 2. [file elife-69523-supp2.docx]

*Key words used in literature retrieval*

A combination of “dyslexia”, “reading disorder”, “reading impairment” or “reading disability” and “fMRI”, “PET”, “voxel-based morphometry”, “VBM” or “neuroimaging” was used in the literature retrieval. Specifically, the combinations of key words were “dyslexia” & “fMRI”; “dyslexia” & “PET”; “dyslexia” & “voxel-based morphometry”; “dyslexia” & “VBM”; “dyslexia” & “neuroimaging”; “reading disorder” & “fMRI”; “ reading disorder” & “PET”; “reading disorder” & “voxel-based morphometry”; “reading disorder” & “VBM”; “reading disorder” & “neuroimaging”; “reading impairment” & “fMRI”; “ reading impairment” & “PET”; “reading impairment” & “voxel-based morphometry”; “reading impairment” & “VBM”; “reading impairment” & “neuroimaging”; “reading disability” & “fMRI”; “ reading disability” & “PET”; “reading disability” & “voxel-based morphometry”; “reading disability” & “VBM”; “reading disability” & “neuroimaging”.
